# Occurrence of yellow fever outbreaks in a partially vaccinated population: An analysis of the effective reproduction number

**DOI:** 10.1371/journal.pntd.0010741

**Published:** 2022-09-15

**Authors:** Fernanda Cristina da Silva Lopes Ferreira, Luiz Antônio Bastos Camacho, Daniel Antunes Maciel Villela

**Affiliations:** 1 National School of Public Health (ENSP), FIOCRUZ, Rio de Janeiro, Brazil; 2 Program of Scientific Computing (PROCC), FIOCRUZ, Rio de Janeiro, Brazil; The University of Texas Medical Branch at Galveston, UNITED STATES

## Abstract

**Background:**

Yellow fever is endemic in Africa and the Americas, occurring in urban or sylvatic environments. The infection presents varying symptoms, with high case-fatality among severe cases. In 2016, Brazil had sylvatic yellow fever outbreaks with more than 11 thousand cases, predominantly affecting the country’s Southeast region. The state of Minas Gerais accounted for 30% of cases, even after the vaccine had been included in the immunization calendar for at least 30 years.

**Methodology and principal findings:**

We applied parameters described in the literature from yellow fever disease into a compartmental model of vector-borne diseases, using namely generation time intervals, vital host and vector parameters, and force of infection, using macroregions as the spatial unit and epidemiological weeks as the time interval. The model permits obtaining the reproduction number, which we analyzed from reported cases of yellow fever from 2016 to 2018 in residents of the state of Minas Gerais, Brazil. Minas Gerais recorded two outbreak periods, starting in EW 51/2016 and EW 51/2017. Of all the reported cases (3,304), 57% were men 30 to 59 years of age. Approximately 27% of cases (905) were confirmed, and 22% (202) of these individuals died. The estimated effective reproduction number varied from 2.7 (95% CI: 2.0–3.6) to 7.2 (95% CI: 4.4–10.9], found in the Oeste and Nordeste regions, respectively. Vaccination coverage in children under one year of age showed heterogeneity among the municipalities comprising the macroregions.

**Conclusion:**

The outbreaks in multiple parts of the state and the estimated *R*_*e*_ values raise concern since the state population was partially vaccinated. Heterogeneity in vaccination coverage may have been associated with the occurrence of outbreaks in the first period, while the subsequent intense vaccination campaign may have determined lower *R*_*e*_ values in the second period.

## Author summary

Yellow fever attracts important research interest since it is an avoidable disease and is still recurrent in Brazil. Although the country has an important public policy that integrates production, distribution, and routine application of the yellow fever vaccine, the disease is still included on the list of endemic infectious diseases in some regions. Surprisingly, regions that were not previously part of risk areas for yellow fever were heavily affected by the outbreak from 2016 to 2018 in the country. Understanding the outbreak’s occurrence and intensity in the state of Minas Gerais based on the effective reproduction number, the focus of this article, is just part of the larger goal of defining with greater certainty the risk areas for yellow fever and proposing measures to control the spread of the disease.

## Introduction

Yellow fever (YF) is an endemic infectious disease in Africa, Central America, and South America. In the Region of the Americas, the disease has mainly two transmission cycles, and even a third one in Africa, which depend on the environment of vector and hosts. In the urban cycle, the important vectors are mosquitoes from genus *Aedes*. Mosquitoes from genera *Sabethes* and *Haemagogus* are common in the sylvatic cycle in South America [1,2]. In this cycle, nonhuman primates (NHPs) are responsible for maintaining the disease in the environment functioning as the main reservoir [3]. The third cycle called the savannah cycle, existing in Africa, involves *Ae*. *simpsoni*, a species that makes the connection between urban and wild cycles [4]. The disease is endemic in Brazil, with sylvatic cases reported recurrently [5]. Thanks to efforts since the 1930s to control the vector in the urban cycle, Brazil has reported no cases of urban yellow fever since 1942 [6]. The country has also established Areas with Recommendation for Vaccination (AWRV) against yellow fever, given the risk of cases of the sylvatic form, which includes Amazonia, the Central-West, and some states and municipalities in the Southeast region. The recommended coverage target is 100% of the population up to one year of age. According to the vaccination schedule of July 2016, an initial dose should be applied at nine months of age, followed by booster doses every ten years. The guideline also includes persons outside this age bracket residing in endemic areas and travelers who intend to visit those areas [7]. The vaccine provides lasting immunity, such that given the shortage of doses in 2017, Brazil started adopting a single dose based on the recommendation by the World Health Organization. In 2019, the National Immunization Program reviewed the single-dose guideline for children under one year and adopted a booster at four years of age, while maintaining the single dose for other age brackets. A new guideline in 2020 expanded the vaccine’s recommendation to all Brazilian municipalities. Since 1998, the municipalities in the state of Minas Gerais have supplied and applied the vaccine according to the Vaccination Calendar of the National Immunization Program (PNI) [8,9]. Besides routine application, the state of Minas Gerais was the target of at least two major vaccination campaigns, in 1999 and 2010, aimed at containing the increasing number of cases reported in the respective periods. Despite these efforts, from 2016 to 2018, Brazil reported at least 11 thousand cases of yellow fever and 900 deaths, and the state of Minas Gerais had about 30% of all the cases [10,11]. That was the largest YF outbreak in the last 80 years, which surprised health authorities and raised questions on the epidemic process involved. For example, the intense mobility of asymptomatic infected individuals could favor the spread of transmission to peri-urban and urban areas [12].

Studies on YF transmission cycles and the characteristics of its natural history provide an important framework in the international scientific literature. Still, the disease remains a current target of discussions and studies, especially due to urban yellow fever’s impact in Africa and the fear of re-urbanization of the disease in the Americas [5,12]. Many gaps still prompt researchers to understand the YF dynamics, despite the current knowledge about yellow fever’s pathophysiology, ecology, and epidemiology. For example, there is a known inverse relationship between the extrinsic incubation period (EIP) and outdoor temperature, decreasing the time needed for vectors to become infectious at temperatures above 25°C [13–15]. The EIP is estimated for *Hg*. *Janthinomys* within 20 to 24 days at an average temperature of 25°C [16,17]. Experiments showed that the variation of ambient temperature between 25°C and 35°C, similar to field conditions, enables transmission to the extent that it reduces the extrinsic incubation period (EIP) to 12 days [18] Such effect applies to both *Aedes aegypti*, the main vector of urban yellow fever, and *Hg*. *Janthinomys*, the vector most found in the epidemic from 2016 to 2018 in the state of Minas Gerais [19]. Higher rainfall and higher air humidity directly influence the increase in the reported YF cases by promoting an adequate environment for the evolutionary cycle of the vectors [13]. In addition to these factors, the diversity and abundance of species of vertebrate hosts species is another factor directly associated with the occurrence of yellow fever [20,21]. Considering the aspects that favor the maintenance of the disease cycle and the spread of the virus in Brazil, the states in the Southeast Region proved to be highly vulnerable to the occurrence of wild YF cases in a spatial analysis based on multicriteria [22]. Kaul *et al*. pointed out the state of Minas Gerais as prone to the occurrence of the disease, identifying it as the only state with records of cases for more than one cycle within the interval of 156 months (2001–2013) [23]. Studies employing molecular epidemiology helped elucidate the virus’ phylogenetics. Two main genotypes have been identified in Brazil (South America I and South America II), with South America I as the most predominant genotype in the country. Based on South America’s genotype I, five lineages were associated with outbreaks in Brazil, 1A, 1B, 1C, 1D, and more recently 1E. A sub-lineage of lineage 1E, identified in samples from humans and primates during the outbreak from 2016 to 2018, was considered responsible for the outbreak in that period [24–26].

Other studies have used mathematical modeling to understand the spread of diseases [27,28]. Given the complexity of transmissible diseases, mathematical models allow simulating situations and conditions that can occur in diverse scenarios, potentially contributing to the elaboration of prevention and control measures [28]. From the perspective of mathematical modeling, an old concept has been an important target of discussion and research, namely the basic reproduction number (*R*_*0*_). Basic reproduction number is defined as the number of secondary cases originating from a primary case in a totally susceptible population [27–31]. For diseases in which part of the population is immune, the effective reproduction number (*R*_e_) is used, with a similar definition to that of *R*_0_, but without assuming the population’s total susceptibility as a requirement for its calculation, thus allowing its estimation when part of the population is considered immune [32].

The knowledge established thus far clarifies and reinforces the spatial and temporal distribution of yellow fever. Minas Gerais is a large and populous state (586,528 km^2^, over 21 million inhabitants) with diverse environmental and sociodemographic elements relevant to YF transmission. It borders endemic regions in the north-west, and states previously free from yellow fever in the east. Our study aims to understand the spatial variation of the effective reproduction number of yellow fever to explain the epidemic recorded in the state of Minas Gerais from 2016 to 2018, considering the preexisting vaccination coverage.

## Material and methods

### Type of study

A combination of a cross-sectional and ecological approach, based on the YF cases reported to the Ministry of Health from 2016 to 2018.

### Data

We selected reported cases of yellow fever in residents of the state of Minas Gerais from all age brackets. The data from the Information System on Diseases of Notification (SINAN) were made available by the Ministry of Health per request. The SINAN database receives data on suspected or confirmed cases of diseases with compulsory notification at the municipal, state, and national levels. The records include information on individuals (date of birth, age, sex, schooling, and place of residence), clinical and laboratory aspects of the disease (symptoms, date of onset, test results), and information on the surveillance process (probable place of infection, occurrence of epizootics, notification date, date of data entry in the system), among other information.

Population data were obtained from IBGE (Brazilian Institute of Geography and Statistics). The state of Minas Gerais has 853 municipalities grouped in 13 macroregions according to the Regionalized Master Plan (PDR-2014), established for organization of services under the Unified Health System (SUS). Minas Gerais population was 19.6 million people in the last official census in 2010. Cases were aggregated according to this division in macroregions according to the patients’ place of residence.

The dates entered in the SINAN database are classified according to the preestablished epidemiological calendar for the entire country, normally consisting of 52 epidemiological weeks. This standardization of dates allowed the selected cases to be grouped according to the epidemiological week at the onset of symptoms, from epidemiological weeks 49/2016 to 52/2018. Cases were grouped by epidemiological week and year of onset of symptoms, with no distinction between confirmed and probable cases. The data on vaccination coverage are available from the DATASUS site (the Ministry of Health’s data website).

Macroregions without outbreaks were excluded from the calculation of *R*_*e*_. Data were analyzed using R software, version 4.0.3.

### Parameters

The model includes entomological parameters and natural history of the disease, namely exponential growth rate of reported cases (*Λ*), extrinsic incubation period (*τ*_*e*_), intrinsic incubation period (*τ*_*i*_) and mosquito survival time (*1/μ*_*m*_). The biology of YF vectors depends on environmental characteristics. Temperature, for example, is determinant for the parameters of mosquito survival time and extrinsic incubation period used in this equation. Thus, we chose to consider the values of the parameters, whenever available in the references consulted, whose temperature of the experiments was in line with the average temperature (25°C) recorded at the end of 2016 and beginning of 2017, in Minas Gerais (National Institute of Meteorology https://portal.inmet.gov.br/dadoshistoricos data).

### Effective reproduction number

The analysis of the effective reproduction number was chosen since yellow fever was endemic in Minas Gerais, which integrates the Area with Recommendation for Vaccination. It was, thus, expected that a large share of the resident population in the state had established some immunity from natural infection or vaccination. The effective reproduction number is thus most recommended, with the advantage of potentially assessing control measures, normally based on the identification of outbreaks [38].

Calculation of the reproduction number requires a model that considers the transmission dynamics of the disease and its specific characteristics. Due to the similarities between dengue and yellow fever, whose vectors belong to the same family (*Culicidae*), the approach here leverages the modeling approach by Villela *et al*. [39] to calculate the basic reproduction number of Zika in the city of Rio de Janeiro in 2015. This approach was based on a model by Pinho *et al*. [40] to describe dengue epidemics in the city of Salvador, Bahia, Brazil, in the years 1995/1996 and 2002. The model considers vector-borne disease transmission and contains vector and human components. There are four mosquito compartments: the mosquito’s aquatic phase and the adult phase subdivided into susceptible, exposed, and infectious. The human portion of the model has compartmental subdivisions susceptible, exposed, infected, and recovered. The model permits to derive the effective reproduction number *R*_*e*_ under an assumption of exponential growth of infected cases. According to Zhao et al. [41], *R*_*e*_ = *R*_0_ (*S*_*h*_/*N*_*h*_) (*S*_*m*_/*N*_*m*_), where *R*_0_ is the basic reproduction number and factors *S*_*h*_/*N*_*h*_ *and S*_*m*_/*N*_*m*_ indicate the ratios between the number of susceptible individuals and total individuals for humans (*h*) and mosquitoes (*m*). In the study by Villela *et al*. [36], Zika was emergent in Rio de Janejro and the whole population was susceptible, permitting to find the basic reproduction number. Here, the number of humans susceptible to YF virus is hard to estimate, since the virus was already circulating in Minas Gerais and population was partly vaccinated. However, during the exponential period of outbreaks, the number of vaccinated individuals is not expected to change significantly and most of vaccinated individuals were immunized prior to the outbreaks. Given this difference between the Zika (or dengue) outbreaks and YF outbreaks, the number *S*_*h*_ excludes vaccinated individuals and the equation below implicitly evaluates the effective reproduction number, given an estimation of the growth rate Λ and the other parameters listed in Table 1:

$$R_{e}=\sqrt{\left(1+\frac{\Lambda }{\mu_{m}}\right)\left(1+\frac{\Lambda }{\gamma }\right)\left(1+\;\frac{\Lambda }{\tau_{e}^{-1}\mu_{m}}\right)\left(1+\frac{\Lambda }{\tau_{i}^{-1}}\right)}$$ (1)

Eq 1 is based on the force of infection according to the increase in cases at the start of the epidemic. The growth rate (Λ) was obtained via log linear regression for each macroregion during the exponential growth period, first chosen by inspection and subsequently evaluated with different intervals of weeks in order to obtain the curve with highest R^2^ (Table A in S2 Text). Considering that a very long period of time could determine a false period of exponential growth, misleading the estimation of the reproduction number, only intervals of 3 to 7 weeks were considered. Given the variability in the other parameters, a Gaussian distribution with the intervals from literature data (Table 1) setting intervals of 2.5% and 97.5% percentiles was applied to each parameter to compose a sample of N = 1000 sets of parameters. The samples were applied in Eq 1 to obtain a resulting sample of estimations of the reproduction numbers. Mean values, standard deviation and uncertainty intervals, 95% confidence intervals, are obtained from these samples. We also applied other methods such exponential growth (EG) [42] and maximum likelihood (ML) [43] to estimate the reproduction number, using overall parameters such as mean generation time and standard deviation. The generation time was considered by summing the intrinsic incubation period of 6 days [1,3] and the average extrinsic incubation period of 12 days [17], totaling 18 days. The transmission times are much shorter than the incubation periods [33]. As the unit of time used was epidemiological week, the total period was divided by 7 days. Thus, the mean generation time was 2.6 weeks, and the standard deviation was 0.5.

**Table 1:** Parameters of the natural history of yellow fever, their definitions and range of values used in the analysis.

Parameters	Definition	Estimate (days)	Reference
IIP	Intrinsic incubation period	4–7	Biggerstaff et al, 2010 [33]
		3–6	Monath, TP, 2001 [1]
		6 (mean)	Vasconcelos, 2002 [3]
Interval used		3 to 7 days	
EIP	Extrinsic incubation period (*Aedes)*	4–18 (12)	Sellards, AW, 1935 [34]
		10–16	Monath, TP, 2001 [35]
		12–16	Biggerstaff et al, 2010 [33]
		12	Bauer & Hudson, 1928 [13]
Interval used		4 to 12 days	
1/ μ_m_	Survival of *Aedes* mosquito	7–10	Bellan SE, 2010 [14]
		3.16–7.48	Maciel-Freitas et al, 2008 [36]
Interval used		3 to 10 days	
EIP	Extrinsic incubation period (*Haemagogus)*	13–15	*Bates* and *Roca*-*Garcia*,*1945* [17]
		12 (25 to 35°C)	
Interval used		12 to 15 days	
1/ μ_m_	Survival of *Haemagogus* mosquito	13.8 to 18.3	Bates, M, 1946 [16]
		7.4 to 20	Mondet, 1997 [37]
Interval used		7.4 to 18.3 days	
Λ	Exponential growth rate of cases	Estimated log-linear regression	

### Vaccination exploratory analysis

The data of vaccination coverage were taken from the DATASUS website. The proportion of vaccinated children in municipalities were categorized into: < 60%, 60–94%, and > 95%. In order to evaluate spatially the macroregions according to vaccination coverage, we also calculated the mean and standard deviation of vaccination coverage per macroregions. The coefficients of variation in the macroregions are obtained from the ratio between the standard deviations to the means of observed coverages within the macro-regions.

Statistical analyses were carried out with R software platform version 4.0.3 (2020-10-10) and the implementation in package R0 [44] was used for methods EG [42] and ML [43].

### Ethical considerations

This study used secondary, anonymized data, thus exempting the study’s submission to a research ethics committee.

## Results

A total of 3,304 YF cases were reported in the state of Minas Gerais from 2016 to 2018. Of the 3,304 reported cases, 72% were males and 56.6% were 30 to 59 years of age (Table 2). The Centro health region included 27% of all reported cases, followed by the Leste and Nordeste regions, with similar percentages (15.9%). Of all the reported cases, 905 were confirmed by both the laboratory criteria and the clinical-epidemiological criteria. Among the confirmed cases, 202 died. Of the 1,109 individuals with information on prior vaccination, 112 had confirmed yellow fever, and 15 died. Case-fatality was 22%, when considering all the confirmed cases, and 13%, with only cases with a vaccination history.

**Table 2:** Likely and confirmed cases of yellow fever according to selected variables, Minas Gerais, Brazil, 2016 to 2018.

	Reported		Confirmed	
Variables	N	%	N	%
***Sex***				
Male	2,380	72.0	772	85.3
Female	924	28.0	133	14.7
***Age bracket (years)***				
9 or less	124	3.7	5	0.5
10 to 29	779	23.6	91	10.1
30 to 59	1,870	56.6	621	68.8
60+	519	15.7	186	20.6
Missing	12	0.4	-	-
***Vaccine***				
No	1,491	45.1	580	64.1
Yes	1,109	33.6	112	12.4
Missing	704	21.3	213	23.5
***Major health region***				
Centro	984	29.8	250	28.3
Nordeste	574	17.4	171	18.9
Leste	573	17.3	130	14.8
Leste do Sul	337	10.2	134	14.4
Sudeste	308	9.3	103	11.4
Centro Sul	215	6.5	58	6.4
Sul	137	4.1	35	3.9
Oeste	71	2.1	9	1.0
Jequitinhonha	42	1.3	7	0.8
Norte	31	0.9	2	0.2
Triângulo do Norte	12	0.4	-	-
Noroeste	10	0.3	-	-
Triângulo do Sul	10	0.3	-	-
	3,304		905	

**Fig 1:**
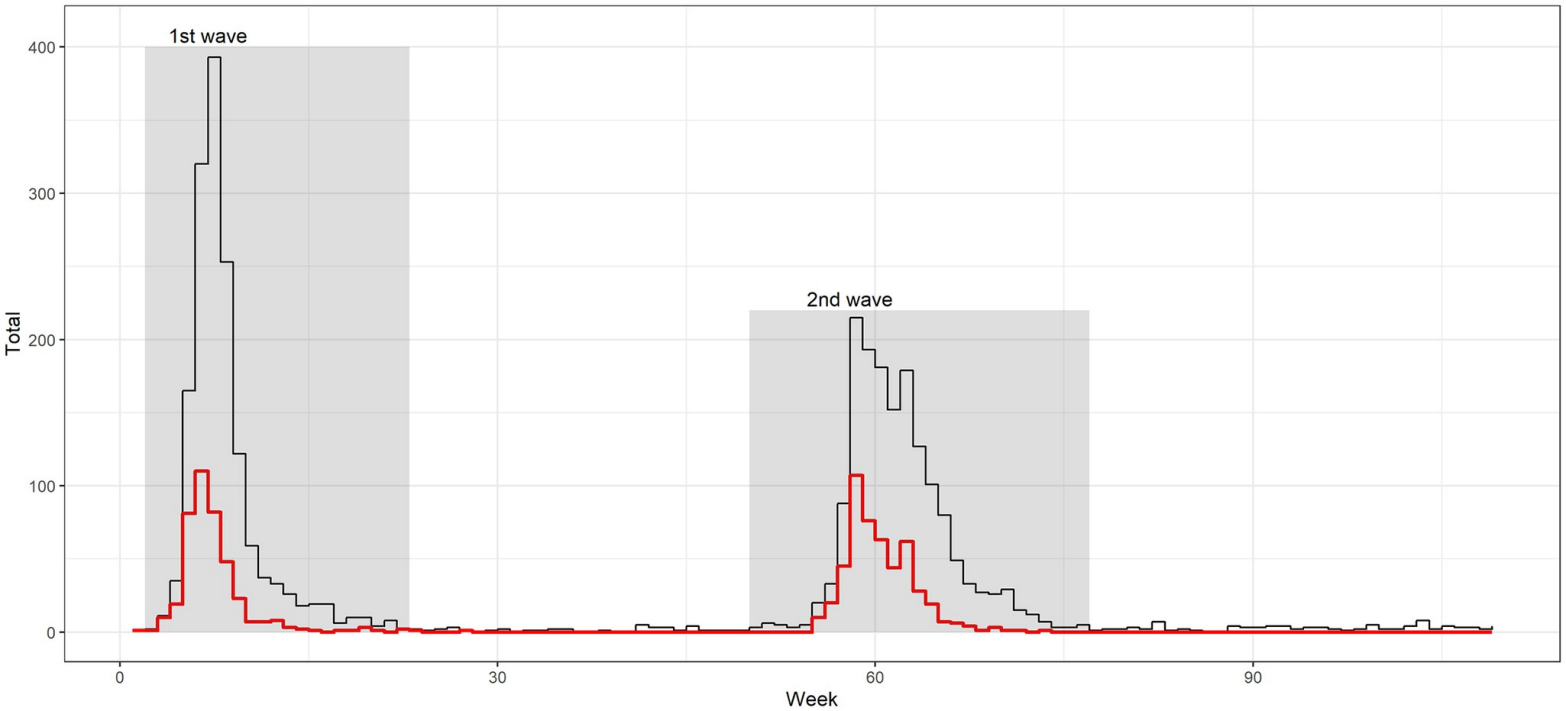
Historical series of reported (black line) and confirmed (red line) yellow fever cases, Minas Gerais, Brazil, 2016–2018. (Epidemiological weeks were transformed into a continuous count with 7-day intervals, totaling 109 weeks).

We identified two YF outbreaks from 2016 to 2018, as evidenced in the time series of cases (Fig 1). The first wave began in December 2016 and reached its peak in January 2017, presenting the widest epidemic curve with the notification of up to 400 cases in one week, totaling 1,554 cases in the period (EW-50/2016 –EW-20/2017). The first cases were reported in the macroregions Nordeste and Leste (EW-50/2016), followed by Leste do Sul (EW-51/2016) and less intensely in the Centro health region, starting in EW-1/2017 (Fig 1). The second wave began in December 2017, reached its peak in January 2018, and showed a lower amplitude compared to the first wave (Fig 1). Despite the second wave’s lower intensity, its duration was longer, having extended from EW-47/2017 to EW-22/2018 and totaling 1,601 reported cases.

**Fig 2:**
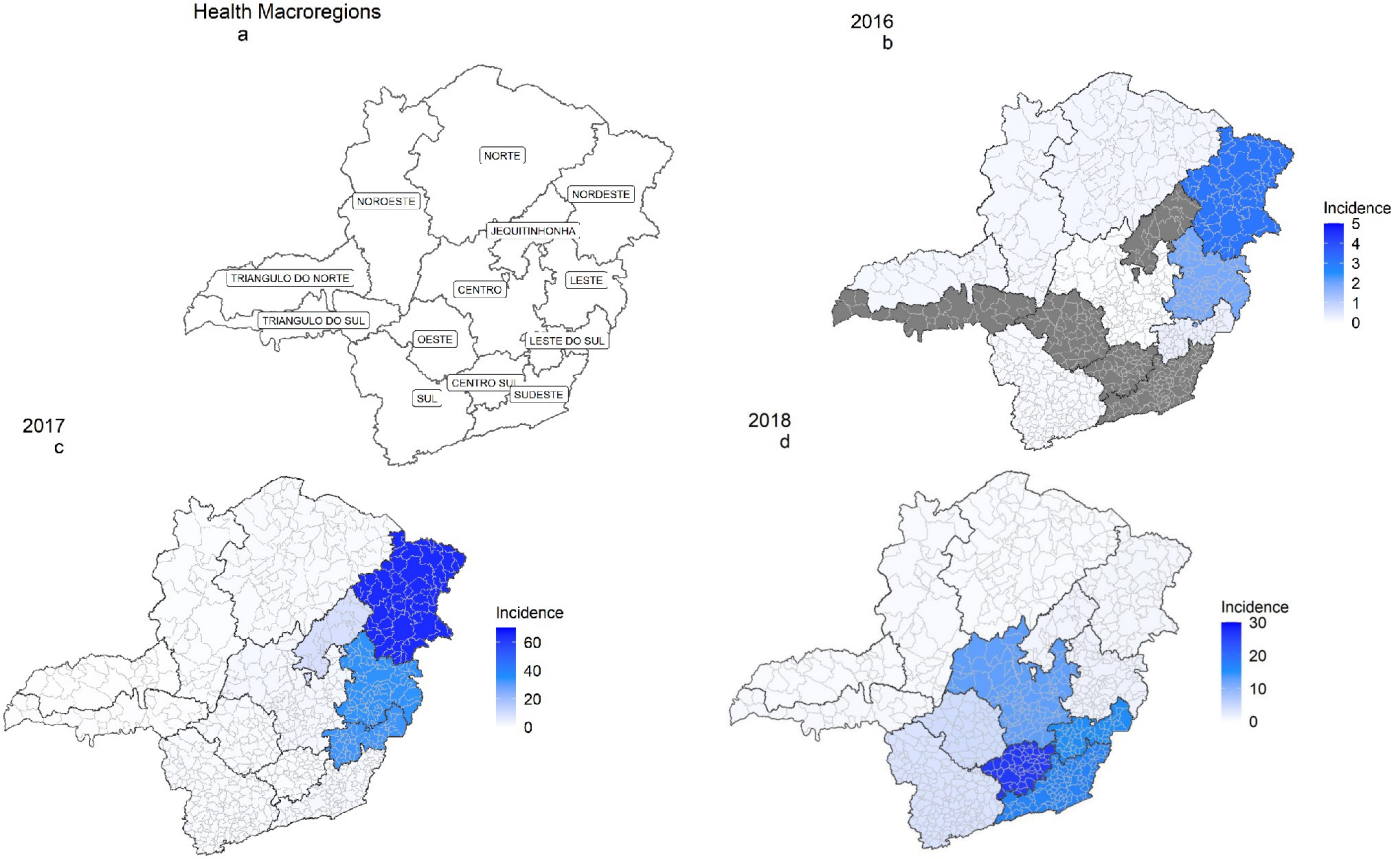
Yellow fever incidence per 100 thousand inhabitants according to major health region and year of onset of symptoms, Minas Gerais, Brazil, 2016 to 2018. Fig **(a)** Minas Gerais state map according to macroregions; **(b)** Incidence of yellow fever per 100,000 inhabitants in 2016, **(c)** Incidence of yellow fever per 100,000 inhabitants in 2017; **(d)** Incidence of yellow fever per 100,000 inhabitants in 2018. Map created with ggplot2 and sf packages (R platform)—base layer from Instituto Brasileiro de Geografia e Estatística (at https://portaldemapas.ibge.gov.br/portal.php#homepage).

The health regions most affected by the second wave were Centro, Centro-Sul, Leste do Sul, Sudeste, and Sul, among which only the Leste do Sul health region presented a recurrence of cases and characterized a new outbreak (Fig 2).

Case incidence per 100 thousand inhabitants was highest in the Nordeste region (3.12) in 2016/2017, in Jequitinhonha (8.8), Leste do Sul (33.0), Leste (34.1), and Nordeste (64.7) regions in 2017/2018, and in Centro Sul (26.3), Sudeste (16.7), Leste do Sul (15.3), and Centro (12.7) in 2018 (Fig 2).

The estimate of the effective reproduction number in the state’s first wave was higher than the one obtained for the second wave (Table 3). All macroregions reported YF cases. The Nordeste and Leste macroregions showed the highest *R*_e_ values in the first wave (Table 3). Reproduction numbers were estimated as high as *R*_e_ = 7.2 (95% CI: 4.4–10.9) for the Nordeste region and estimated at 7.3 (95% CI: 5.2–9.8) for the first wave in the state. Reproduction numbers are larger with *Haemagogus* parameters than those with parameters of *Aedes* mosquitoes.

**Table 3:** Effective reproduction number and strength of infection according to major health region and epidemiological week, Minas Gerais, Brazil, 2016 to 2018. Three- to seven-week periods were selected with increases in cases, normally identified at the start of the outbreak in each of the macroregions. The macroregions in the bold face presented statistically significant values (p value < 0.05) in the regression that estimated the rate of growth of cases. The macroregions Norte, Jequitinhonha, Triângulo do Norte, and Triângulo do Sul did not display upward curves, thus preventing calculation of the reproduction number in these regions.

State of Minas Gerais and health regions	EW*	ʌ**	*R*^*2*^ *statistic*	*p value*	*R*_*e*_ ***	95% CI	*R*_*e*_ ***	95% CI
					*Aedes*		*Haemagogus*	
Minas Gerais								
1^st^ wave	51/16–03/17	1.1	**0.9586**	**p < 0.001**	**5.1**	**3.7–7.0**	**7.3**	**5.2–9.8**
2^nd^ wave	51/17–03/18	0.87	**0.9854**	**p < 0.001**	**4.1**	**3.3–5.3**	**5.6**	**4.6–7.1**
**Centro**	**49/17–03/18**	**0.90**	**0.9039**	**p < 0.001**	**4.3**	**3.2–5.5**	**5.9**	**3.9–8.4**
Centro Sul	02/18–05/18	0.83	0.662	0.120	6.9	1.5–20.3	14.5	1.5–54.2
**Leste**	**50/16–03/17**	**1.01**	**0.909**	**0.002**	**4.7**	**3.6–6.4**	**6.6**	**3.9–10.0**
Leste do Sul	01/17–04/17	1.00	0.9438	0.107	6.1	1.6–14.6	10.8	1.8–33.8
**Nordeste**	**50/16–04/17**	**1.09**	**0.9163**	**0.0017**	**5.0**	**3.7–6.9**	**7.2**	**4.4–10.9**
Norte	02/17–07/17	0.35	0.9809	0.062	2.3	1.5–2.6	2.8	1.4–5.1
**Oeste**	**51/17–04/18**	**0.36**	**0.854**	**0.002**	**2.3**	**1.7–3.4**	**2.7**	**2.1–3.6**
**Sudeste**	**52/17–04/18**	**0.74**	**0.8619**	**0.015**	**3.6**	**2.3–5.3**	**4.9**	**2.4–8.1**
Sul	01/18–05/18	0.86	0.7657	0.081	3.7	1.7–8.4	6.6	1.7–15.4

*epidemiological week;

** growth rate (Λ);

***Effective reproduction number

The reproduction numbers estimated using the EG and ML methods are shown in Table A in S2 Text. These estimations are generally in agreement with values and 95% CI presented in Table 3, with Sudeste as an exception.

**Fig 3:**
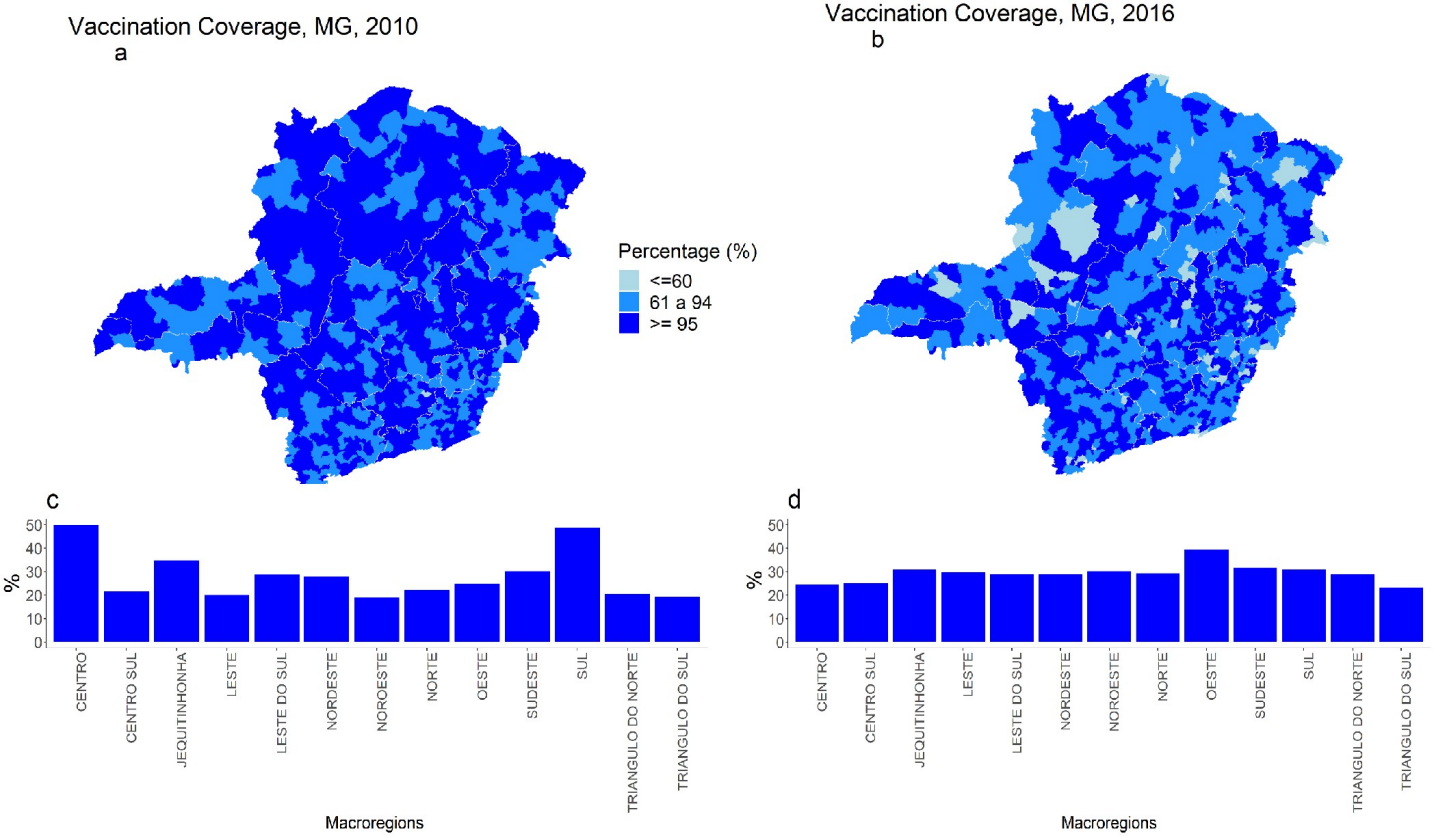
Yellow fever vaccination coverage in children under one year of age according to macroregions, Minas Gerais, Brazil, 2010 and 2016. Vaccination coverage was consulted on the website of the Brazilian Ministry of Health (DATASUS). The data refer to vaccination coverage in children under one year of age and classified in three categories, less than 60%, 61% to 94%, and 95% or greater. Fig (**a)** vaccination coverage in children under one year of age in 2010; **(b)** vaccination coverage in children under one year of age in 2016; **(c)** coefficient of variation in vaccination coverage in 2010; **(d)** coefficient of variation in vaccination coverage in 2016. Map created with ggplot2 and sf packages (R platform)—base layer from Instituto Brasileiro de Geografia e Estatística (https://portaldemapas.ibge.gov.br/portal.php#homepage).

The analysis of YF vaccination coverage in children under one year of age in 2016 showed relevant heterogeneity among the municipalities comprising the macroregions in the state of Minas Gerais (Fig 3). In 2016, we found a predominance of areas with vaccination coverage below 95% and municipalities with even lower coverage, less than 60%, compared to 2010. In 2010, only two of the 853 municipalities in Minas Gerais had a coverage rate of less than 60%, while in 2016, there were 60 such municipalities. The coefficient of variation in vaccination coverage showed homogeneity between the macroregions, but greater heterogeneity between the municipalities in 2016 (Fig 3).

**Fig 4:**
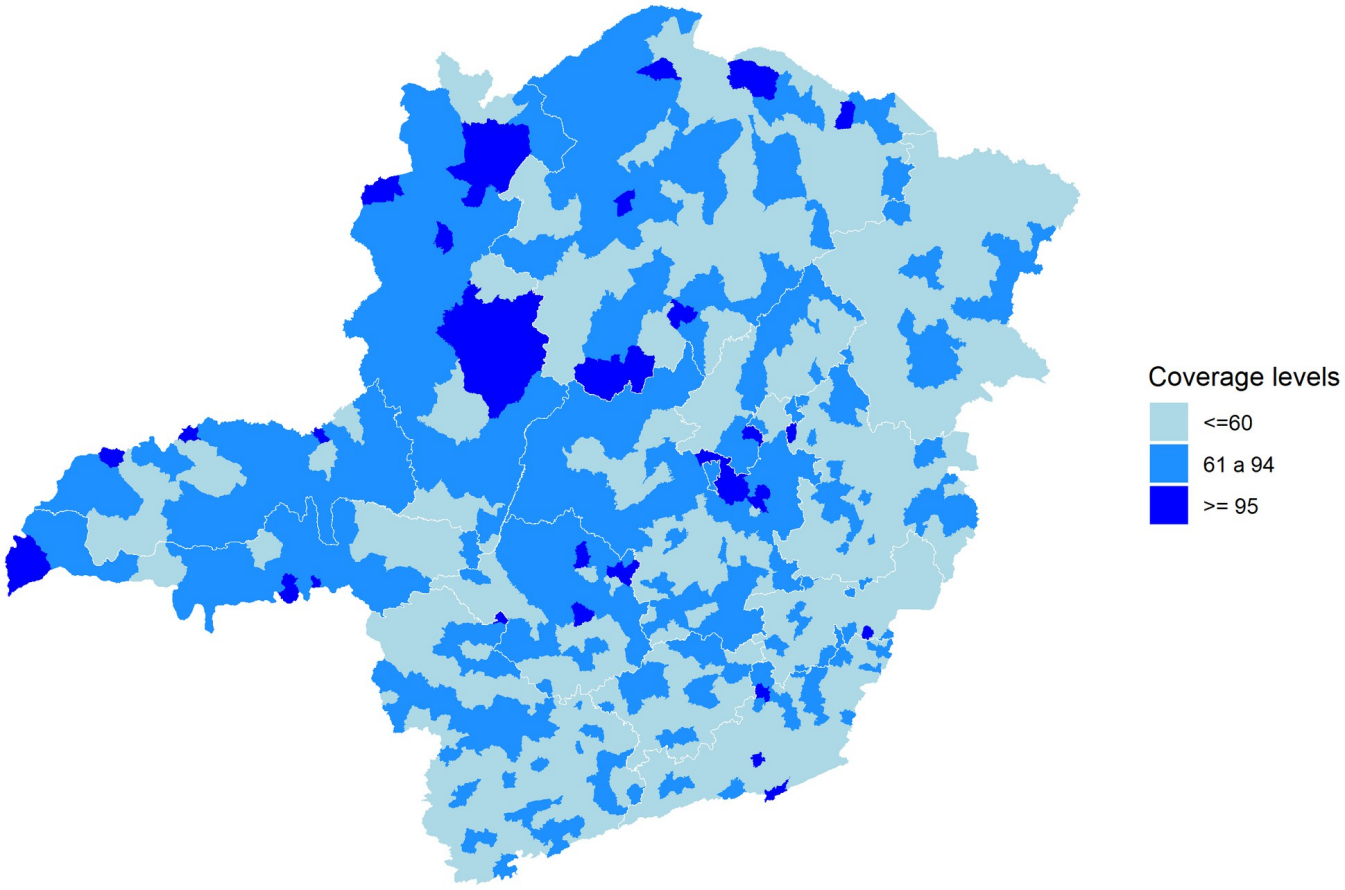
Yellow fever vaccination coverage (2016) in municipality general populations (adult and children). Data from Secretariat of Health of Minas Gerais, Brazil. Coverage categories refer to vaccination coverage in children under one year of age and classified in three categories, less than 60%, 61% to 94%, and 95% or greater. Map created with ggplot2 and sf packages (R platform)—base layer from Instituto Brasileiro de Geografia e Estatística (https://portaldemapas.ibge.gov.br/portal.php#homepage).

The vaccine coverage data show 55% of municipalities with less than 60% of coverage in the general population (Fig 4) while in children under 1 year of age it was 6%. The proportion of municipalities with coverage percentage above 95% was 51% in the age group of children under 1 year of age, while in the general population this proportion was 3%.

## Discussion

The estimation of reproduction numbers varied across regions from 2.7 (95% CI: 2.0–3.6) to 7.2 (95% CI, 4.4–10.9). The estimated figures show the intensity of the outbreaks in an area in which we had a partially vaccinated population. Data from these outbreaks show that cases were detected and confirmed in a few vaccinated individuals. Only a few municipalities had vaccine coverage higher than 95% in the general population. After the first cases in the year 2016, an intense YF vaccination campaign was launched, which our results suggest a positive impact on the number of cases in late 2017 but still did not avoid another wave of cases. The vaccine coverage had to be higher in several places to increase the local immunization and reduce the heterogeneity that revealed spots with lower coverage levels among infants. These results raise concerns, given the availability of a safe, effective, and free vaccine, for at least thirty years.

Case incidence was highest in the macroregions Nordeste and Leste in early 2017, giving these regions the highest *R*_e_ values, with 7.2 (95% CI: 4.4–10.9) and 6.6 (95% CI: 3.9–10.0), respectively. In 2018, the Central macro-region had the highest *R*_e_ = 5.9 (95% CI: 3.9–8.4). These results reinforce the yellow fever spillover from the east side of the state of Minas Gerais [25,26]. During that period the number of weekly reports reached 400 cases, a value higher than the accumulated number of cases for a period of one year at least in the last 30 years [12].

The reproduction numbers estimated with the parameters of the *Haemagogus* wild cycle vector (EIP 22 days and survival time 18.3 days) for macroregions Oeste, Leste, Nordeste e Norte are similar to the values estimated by the EG [42] and ML [43] methods implemented by *R0 Package* (see Fig A to L in S3 Text). The exception was observed for the Centro and Sudeste macroregions, which presented more discrepant values among the applied methods. The estimation for the Centro macroregion was *R*_e_ = 5.9 (95% CI: 3.9–8.4), whereas using methods EG and ML was 10.9 and 8.9, respectively (see Table A in S3 Text). In the Sudeste macroregion, the difference was even greater, since the estimation with Equation (Eq 1) was 4.87 (95% CI: 2.4–8.1), but much higher with other methods 20.3 (95% CI: 8.1, 55.7) (EG) and 11.5 (95% CI: 7.5, 16.7) (ML) (see Table A in S3 Text).

We observed two epidemic curves in the period analyzed in the state of Minas Gerais. The spread of the disease started in the Nordeste health region of the state, which showed the highest case incidence, and continued to the health regions located in the south and traversing the eastern side of the state of Minas Gerais, which borders other states of Southeast Brazil, namely Espírito Santo, Rio de Janeiro, and São Paulo. Given the path of the disease, the state of Minas Gerais has functioned as a transition zone between Central-West Brazil, where yellow fever has been endemic, and the coastal areas of Southeast Brazil for the spread of yellow fever. The second wave of cases began in December 2017, with an increase in reports in the macroregions Centro, Oeste, Sudeste, Centro Sul, and Sul in the state of Minas Gerais and case reports in the states of Espírito Santo, Rio de Janeiro, and São Paulo. Phylogenetic analyses of the viruses found in nonhuman primates and humans that evolved to death after confirmation of yellow fever concluded that the disease spread from the state of Minas Gerais to densely populated regions in the states of São Paulo and Rio de Janeiro [45–47]. The epidemic seemed to have followed a path related to epizootics and clusters of susceptible individuals living, working or playing near forested areas.

The epidemiological weeks identified here with increased cases are consistent with the seasonality of yellow fever in Brazil. The spatial and temporal distribution of yellow fever coincides with heavier rainfall and higher temperatures, providing an optimal environment for the emergence of cases [23]. Such dynamics happen because the increase in temperature drives the vector’s survival and infectiousness, and its abundance increases with rainfall [20]. In this study, Childs *et al*. [20] formulated an environmental risk model for the spread of yellow fever to find different seasonality among Brazil’s five major geographic regions, finally considering the Southeast of Brazil as seasonally adequate for the spread of the disease.

The epidemic showed greater intensity and higher values in the number of effective reproduction in the first wave, compatible with the greater amplitude of the curve, consistent with low vaccination coverage and higher exposures to the wild transmission cycle. As transmission decreased, possibly due to the intense vaccination campaign that started after the confirmation of the first cases, the strength of the infection decreased, tending to decrease the values of *R*_e_ in the second wave. The values of *R*_e_ found in this study, when considered the vector of the urban cycle of the disease, varied from 2.4 a 5.0 (*Aedes*), in agreement with Massad *et al*., that evaluated reproduction numbers for urban yellow fever in the range of 1.35 to 4.21 for outbreaks in São Paulo from 1991 to 2000 [48].

Yellow fever is also highly prevalent in African countries as one of the continent’s most relevant public health problems. In 2015, there were more than seven thousand reported cases of urban yellow fever in Angola. In order to understand the epidemic’s intensity, Zhao *et al*. [41] used a SIR model (susceptible-infected-recovered) to analyze the relationship between *R*_*0*_ and *R*_e_. As occurred in the current study, the epidemic in Luanda, Angola, presented two waves. In the first wave, recorded in January 2016, the estimated effective reproduction number was 4.4 and 5.5. In Luanda’s second wave, five months later, the number *R*_e_ was 2.0 points lower, also attributed to the vaccination campaign [41]. Vaccine campaigns reduce the number of susceptible people, reducing the strength of the disease’s infection [27,32,49]. Another study in Luanda proposed a logistic model to infer the risk of infection with the YF virus and estimated the reproduction number at 4.8. The authors found that population density was the main predictor of the risk of disease. The model showed that the spread of the virus was influenced by urban mobility and the vector’s adaptation to certain districts. Based on the findings, it would be possible to predict areas with increased risk of yellow fever and to target interventions, for example, when there are limited stocks of vaccines [50].

Vaccination is essential for the prevention and control of the disease not only in urban environments but also in the sylvatic form due to the difficulty of eradicating the sylvatic vectors. A study showed that vaccination averted 5.1 times the number of deaths (73) and 5.8 times the number of observed cases (941) in Luanda’s outbreak of December 2015. The reproduction number varied from 1.0 to 8.5 during the 37 weeks of the Luanda epidemic, with the highest values initially (5.0 to 8.5) before mass vaccination of the population started [51]. Another study that assessed the impact of dose sparing for vaccination in Luanda estimated *R*_e_ between 5.2 and 7.1, concluding that maintaining high coverage rates was more important than using full doses [52].

The vaccine coverage in Minas Gerais’ general population is far below the vaccination coverage observed in children younger than one year. In the study by Shearer et al. [53], one of the scenarios evaluated that 97% of the 853 municipalities would have vaccinated between 60 and 94% of the population. Adults are more exposed to the virus and sylvatic YF vectors, both at work and in leisure-time activities. More studies are needed to obtain vaccination coverage in the adult population, especially age-stratified coverages, which could be used to provide a better assessment of the transmission dynamics in these regions. Lack of vaccination coverage is likely the main determinant of susceptibility, given the relatively low incidence of yellow fever in recent decades. The increasing number of unvaccinated individuals in successive birth cohorts provided the pool of susceptibles that led to the epidemic. Therefore, lack of vaccination, rather than vaccine failures, which had low frequency, appeared as a more plausible scenario in that epidemic.

The health surveillance system in Brazil has capillarity, standardized standards, and recommendations that determine the actions to be performed in all health units, public or private. The general guidelines include a list of mandatory diseases by the Ministry of Health, which includes yellow fever. However, underreporting or underdiagnosing the disease is still possible, especially given the broad spectrum of YF symptoms. A common criterion for directing case investigation to other diseases is whether a case was prior immunized with the YF vaccine. Also, asymptomatic infections might happen, with previous estimations of about 55% of infected people without symptoms and 33% with mild form [54]. No significant bias is expected in the estimation of the effective reproduction number when underreporting is time-invariant. However, improvements in surveillance are more likely to occur at the very beginning of the epidemic and to stabilize soon in the first wave. In the second wave, less underreporting and less variation are expected, as surveillance was already enhanced. In fact, analysis with only confirmed cases reveals similar estimations of the effective reproduction number.

A few limitations in the design and methodology might impact the assessment of the reproduction number. Entomological parameters still carry uncertainties since studies with parameters of wild vectors are still scarce, given the difficulty of creating Neotropical wild mosquitoes in the laboratory [46,47]. Also, the base model used in this work refers to the cycle of dengue transmission, which does not include an intermediate host as occurs in wild yellow fever. Furthermore, more data and studies on age-stratified vaccination coverage could help to better understand the current epidemiology of yellow fever. Moreover, the mathematical framework does not include an explicit compartment for vaccinated individuals. However, the estimation is based on short time scales, i.e., during the exponential period of outbreaks, and realized with a fair assumption that most vaccinated individuals were immunized prior to the outbreak.

The outbreaks of yellow fever recorded in Brazil from 2016 to 2019 instigated researchers and the scientific community to understand the occurrence of many YF cases, severe cases, and deaths in a state with a large, partially vaccinated population. The results in this work show the intensity of the outbreaks requiring an even higher coverage of YF vaccination in the state and the need for studies of YF revaccination. The lessons learned from that epidemic should be applied to other regions, such as Northeastern states, which did not have YF outbreaks. Such considerations can also apply to other endemic areas in other countries. Finally, it remains important to monitor the risk of urban yellow fever outbreaks constantly.

## Supporting information

S1 Text Equation derivation under assumption of exponential growth. (PDF)

S2 Text Identification and selection of exponential case growth period. Table A. Results of the evaluation of the exponential period with the variation of the aggregation window of notified cases and their respective values of p and R^2^ according to location (State and Macroregions of Minas Gerais). Fig A—Curve of reported cases for Minas Gerais and the highlight for the exponential growth period for 1st wave and 2nd wave. Fig B—Curve of reported cases for Macroregion Centro and the highlight for the exponential growth period. Fig C—Curve of reported cases for Macroregion Centro Sul and the highlight for the exponential growth period. Fig D—Curve of reported cases for Macroregion Leste and the highlight for the exponential growth period. Fig E—Curve of reported cases for Macroregion Leste do Sul and the highlight for the exponential growth periodFigure F—Curve of reported cases for Macroregion Nordeste and the highlight for the exponential growth period. Fig G—Curve of reported cases for Macroregion Norte and the highlight for the exponential growth period. Fig H—Curve of reported cases for Macroregion Oeste and the highlight for the exponential growth period. Fig I—Curve of reported cases for Macroregion Sudeste and the highlight for the exponential growth period. Fig J—Curve of reported cases for Macroregion Sul and the highlight for the exponential growth period.
(PDF)

S3 Text Different methods of estimating the reproductive number. Fig A - 1st Wave of reported cases of yellow fever in the state of Minas Gerais and period selected to estimate reproductive number by the Methods EG and ML. Fig B—2nd Wave of reported cases of yellow fever in the state of Minas Gerais and period selected to estimate reproductive number by the Methods EG and ML. Fig C—Reported cases of yellow fever in the Macrorregion Centro state of Minas Gerais and period selected to estimate reproductive number by the methods EG and ML. Fig D—Reported cases of yellow fever in the Macrorregion Centro Sul state of Minas Gerais and period selected to estimate reproductive number by the methods EG and ML. Fig E—Reported cases of yellow fever in the Macrorregion Leste state of Minas Gerais and period selected to estimate reproductive number by the methods EG and ML. Fig F—Reported cases of yellow fever in the Macrorregion Leste do Sul state of Minas Gerais and period selected to estimate reproductive number by the methods EG and ML. Fig G—Reported cases of yellow fever in the Macrorregion Nordeste state of Minas Gerais and period selected to estimate reproductive number by the methods EG and ML. Fig H—Reported cases of yellow fever in the Macrorregion Norte state of Minas Gerais and period selected to estimate reproductive number by the methods EG and ML. Fig I—Reported cases of yellow fever in the Macrorregion Oeste state of Minas Gerais and period selected to estimate reproductive number by the methods EG and ML. Fig J—Reported cases of yellow fever in the Macrorregion Sudeste state of Minas Gerais and period selected to estimate reproductive number by the methods EG and ML. Fig L—Reported cases of yellow fever in the Macrorregion Sul state of Minas Gerais and period selected to estimate reproductive number by the methods EG and ML. Table A. Effective number of yellow fever reproduction according to estimation method (Exponential Growth e Maximum Likelihood), Minas Gerais, Brazil, 2016 to 2018.
(PDF)
