# Supplementary material for: Occurrence of yellow fever outbreaks in a partially vaccinated population: An analysis of the effective reproduction number
Source: PLoS Negl Trop Dis. 2022 Sep 15;16(9):e0010741. doi: 10.1371/journal.pntd.0010741 (PMC9514630; doi:10.1371/journal.pntd.0010741)
Supplement: S1 Text — (PDF) [file pntd.0010741.s001.pdf]

Supplementary Material S1:

Occurrence of yellow fever outbreaks in a partly vaccinated population: an analysis of the effective reproduction number

Authors and affiliations
Fernanda Cristina da Silva Lopes Ferreira[1], Luiz Antônio Bastos Camacho[1], Daniel Antunes Maciel Villela[2*]

[1] National School of Public Health (ENSP), FIOCRUZ, Rio de Janeiro, Brazil
[2] Program of Scientific Computing (PROCC), FIOCRUZ, Rio de Janeiro, Brazil

Corresponding author. Email: daniel.villela@fiocruz.br

S1 Equation derivation

The model requires an assumption of exponential growth over a time interval.
Thus, over this particular period: $I(t) = I_0 \exp(\Lambda t)$.

The derivative is given by: $\frac{dI(t)}{dt} = I_0 \Lambda \exp(\Lambda t)$.

In the case of a SIR model, replacing the right hand side in the equation related to the infected compartment permit to obtain $\Lambda/\gamma = (\beta/\gamma) * S/N - \Lambda$, where S/N is the fraction of susceptible individuals in the population. Therefore, $R_e = R_0(S/N) = (\beta/\gamma)(S/N) = \Lambda(1 + 1/\gamma)$. The effective reproduction number is given by the reproduction number and a multiplicative factor given by the proportion of susceptible individuals.

In the same fashion, using Next Generation method (NGM), Zhao et al [1]. showed that the effective reproduction number of yellow fever, as a mosquito-vector disease, is quantity scaled after the proportion given by two factors, namely the proportion of susceptible mosquitoes and susceptible humans.

In other words, from Zhao et al, with and adjustment as noted below:
$R_e = R_0(S_h/N_h)\ (S_m/N_m)$.

We should note that, since Zhao et al consider the NGM, the method finds the R_0 and R_e per compartment. For this reason, the equation has a square root of these proportions. Here, in our work we considered the number of human cases in a generation after the previous cases in human. Therefore, Equation (1) in the methodology provides an expression for Re. In the beginning of an epidemic, $S_h = N_h$ and $S_m = N_m$, resulting in R_0.
Since the population of the study was partly vaccinated and yellow fever is endemic, the method described in the methodology provides estimates of R_e.

References

1. Zhao S, Musa SS, Hebert JT, Cao P, Ran J, Meng J, et al. Modelling the effective reproduction number of vector-borne diseases: the yellow fever outbreak in Luanda, Angola 2015–2016 as an example. PeerJ. 2020;8: e8601. doi:10.7717/peerj.8601
