# Supplementary material for: Occurrence of yellow fever outbreaks in a partially vaccinated population: An analysis of the effective reproduction number
Source: PLoS Negl Trop Dis. 2022 Sep 15;16(9):e0010741. doi: 10.1371/journal.pntd.0010741 (PMC9514630; doi:10.1371/journal.pntd.0010741)
Supplement: S2 Text — Table A. Results of the evaluation of the exponential period with the variation of the aggregation window of notified cases and their respective values of p and R2 according to location (State and Macroregions of Minas Gerais). Fig A—Curve of reported cases for Minas Gerais and the highlight for the exponential growth period for 1st wave and 2nd wave. Fig B—Curve of reported cases for Macroregion Centro and the highlight for the exponential growth period. Fig C—Curve of reported cases for Macroregion Centro Sul and the highlight for the exponential growth period. Fig D—Curve of reported cases for Macroregion Leste and the highlight for the exponential growth period. Fig E—Curve of reported cases for Macroregion Leste do Sul and the highlight for the exponential growth periodFigure F—Curve of reported cases for Macroregion Nordeste and the highlight for the exponential growth period. Fig G—Curve of reported cases for Macroregion Norte and the highlight for the exponential growth period. Fig H—Curve of reported cases for Macroregion Oeste and the highlight for the exponential growth period. Fig I—Curve of reported cases for Macroregion Sudeste and the highlight for the exponential growth period. Fig J—Curve of reported cases for Macroregion Sul and the highlight for the exponential growth period. (PDF) [file pntd.0010741.s002.pdf]

Supplementary Material S2:

Occurrence of yellow fever outbreaks in a partly vaccinated population: an analysis of the effective reproduction number

Authors and affiliations

Fernanda Cristina da Silva Lopes Ferreira[1], Luiz Antônio Bastos Camacho[1], Daniel Antunes Maciel Villela[2*]

[1] National School of Public Health (ENSP), FIOCRUZ, Rio de Janeiro, Brazil

[2] Program of Scientific Computing (PROCC), FIOCRUZ, Rio de Janeiro, Brazil

Corresponding author. Email: daniel.villela@fiocruz.br

S2 Text Identification and selection of the period of case growth

Table A in S2 Text. Results of the evaluation of the exponential period with the variation of the aggr egation window of notified cases and their respective values of p and R² according to location (State and Macroregions of Minas Gerais).

	Coeficient	N. SE	Week	R^2	*P_value*	R_e (Aedes)*	R_e (haemagogus)**
Minas Gerais (1st wave)	1.09 (0.72,1.45)	6	2 a 7	0.93	0.001154	3.91	6.03
	**1.1 (0.86,1.34)**	**7**	**1 a 7**	**0.9586**	**7.617e-05**	**3.95**	**6.11**
	0.85 (0.43,1.27)	7	2 a 8	0.8126	0.003472	3.12	4.06
Minas Gerais (2nd wave)	0.90040 (0.69,1.11)	5	54 a 58	0.9781	0.0008975	3.27	4.89
	**0.87042 (0.74,1)**	**6**	**53 a 58**	**0.9854**	**5.112e-05**	**3.18**	**4.80**
	0.75348	6	54 a 59	0.9245	0.00140	2.87	4.08
Centro	**0.9055 (0.6,1.21)**	**7**	**52 a 58**	**0.9039**	**0.0006354**	**3.29**	**4.92**
			51 a 58	Não converge			
	0.8168 (0.57,1.07)	8	52 a 59	0.8988	0.0002111	3.01	4.42
	0.8883 (0.56,1.21)	7	53 a 59	0.8899	0.0008958	3.23	4.82
Centro Sul	**0.8287 (-0.53,2.19)**	**4**	**57 a 60**	**0.662**	**0.1199**	**3.05**	**4.48**
		5	56 a 60	Não converge			
	0.5619 (-0.2,1.32)	5	57 a 61	0.5316	0.09998	2.27	3.12
	0.3935 (-0.12,0.9)	6	57 a 62	0.4166	0.09931	1.84	2.38
Leste	**1.0087 (0.62,1.40)**	**6**	**1 a 7.5**	**0.909**	**0.002038**	**3.63**	**5.54**
	0.7825 (0.36,1.21)	7	1 a 8	0.7829	0.00507	2.90	4.23
	0.5887 (0.12,1.06)	6	2 a 8	0.686	0.02598	2.34	3.25
Leste do Sul	**1.0037 (-1.16,3.17)**	**3**	**5 a 7.5**	**0.9438**	**0.1072**	**3.62**	**5.51**
	0.53295(-0.68,1.75)	4	4 a 8	0.4608	0.1997	2.19	2.99
Nordeste	**1.0863 (0.68,1.49)**	**6**	**2 a 7.5**	**0.9163**	**0.00172**	**3.90**	**6.02**
			1 a 8	Não converge			
	0.59802 (0.13,1.07)	8	2 a 9	0.556	0.02046	2.37	3.29
	0.9886 (0.34,1.64)	5	3 a 7	0.8479	0.01696	3.56	5.41

Norte	0.2485 (0,0.5)	4	6 a 9.5	0.8497	0.05142	1.50	1.81
			5 a 9.5	Não converge			
	**0.35 (-0.09,0.78)**	**3**	**7 a 9.5**	**0.9809**	**0.06226**	**1.72**	**2.19**
	0.06931 (-0.28,0.42)	5	6 a 10	-0.1746	0.5695	1.13	1.21
Oeste	0.3813 (0.15,0.61)	6	54 a 59	0.7982	0.01036	1.81	2.33
	**0.3618 (0.21,0.52)**	**7**	**53 a 59**	**0.854**	**0.001834**	**1.76**	**2.25**
			52 a 59	Não converge			
	0.3214 (-0.05,0.69)	5	55 a 59	0.6237	0.07003	1.66	2.09
Sudeste	**0.7398 (0.28,1.2)**	**5**	**55 a 59**	**0.8619**	**0.01462**	**2.77**	**4.00**
			54 a 59	Não converge			
	0.8651 (-0.07,1.8)	4	56 a 59	0.8309	0.05807	3.16	4.69
	0.5853 (0.22,0.95)	6	54 a 60	0.7931	0.0109	2.33	3.23
Sul	**0.861 (-0.27,1.99)**	**4**	**56 a 59.5**	**0.7657**	**0.0814**	**3.15**	**4.67**
	0.5006 (-0.32,1.32)	5	56 a 60	0.4107	0.1468	2.11	2.84
			55 a 60	Não converge			

Figures A to J in S2 Text shows on the left the curve of reported cases for each site (State and Macro-region of Minas Gerais) throughout the period of the yellow fever epidemic (2016 to 2019) and on th e right the highlight for the exponential growth period for regression application in order to estimate t he number of reproductive.

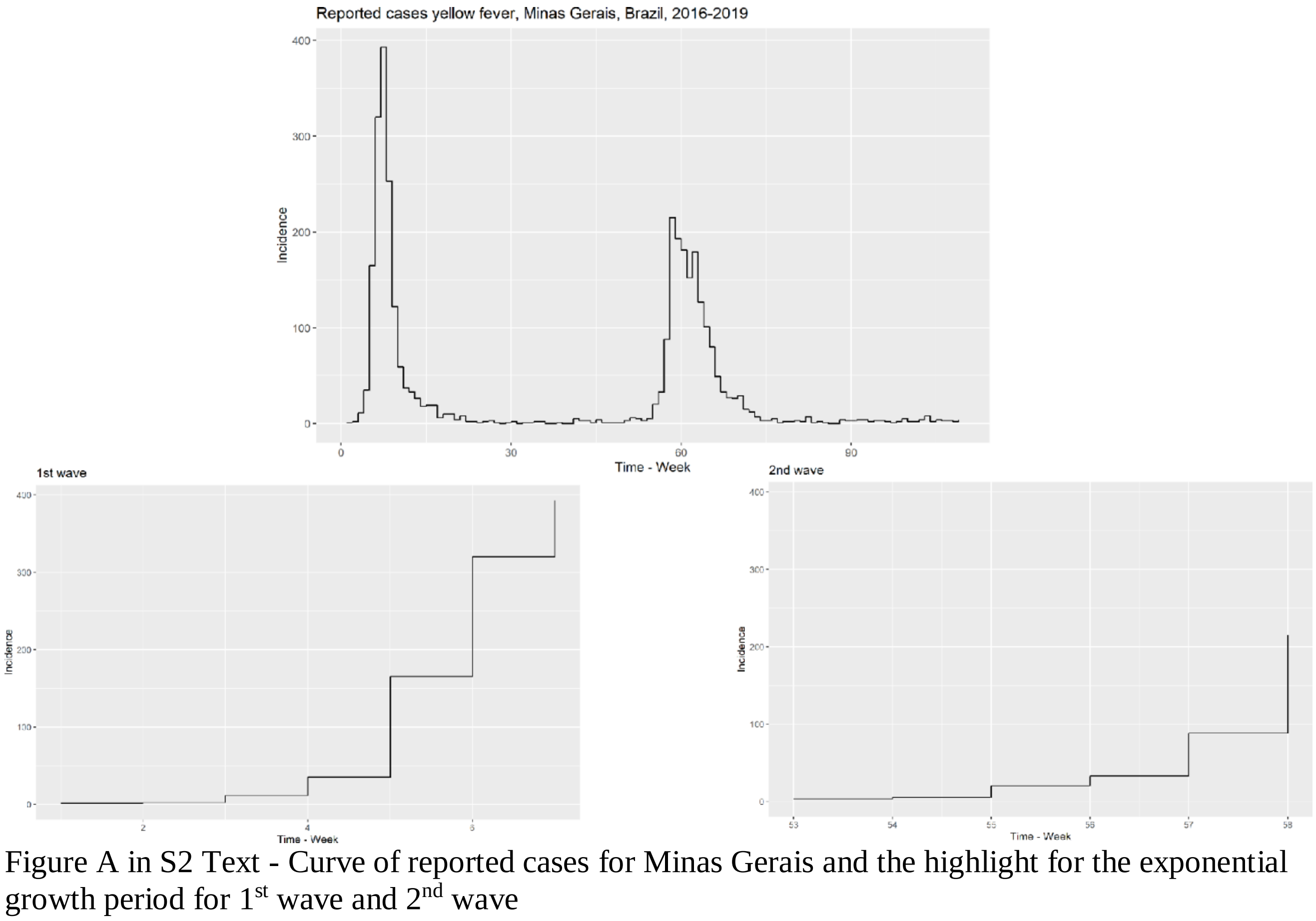

Figure A in S2 Text - Curve of reported cases for Minas Gerais and the highlight for the exponential growth period for 1st wave and 2nd wave

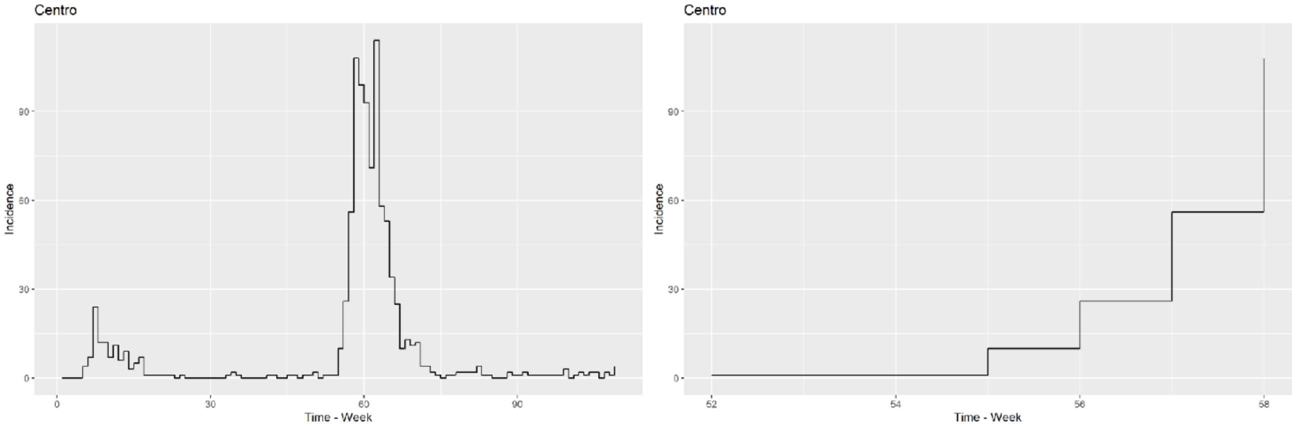

Figure B in S2 Text - Curve of reported cases for Macroregion Centro and the highlight for the exponential growth period

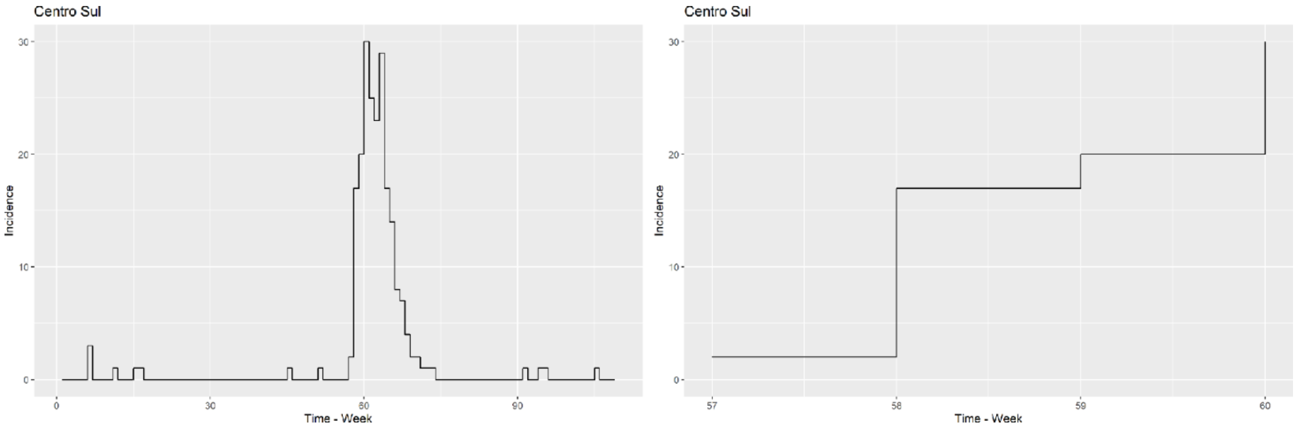

Figure C in S2 Text - Curve of reported cases for Macroregion Centro Sul and the highlight for the exponential growth period

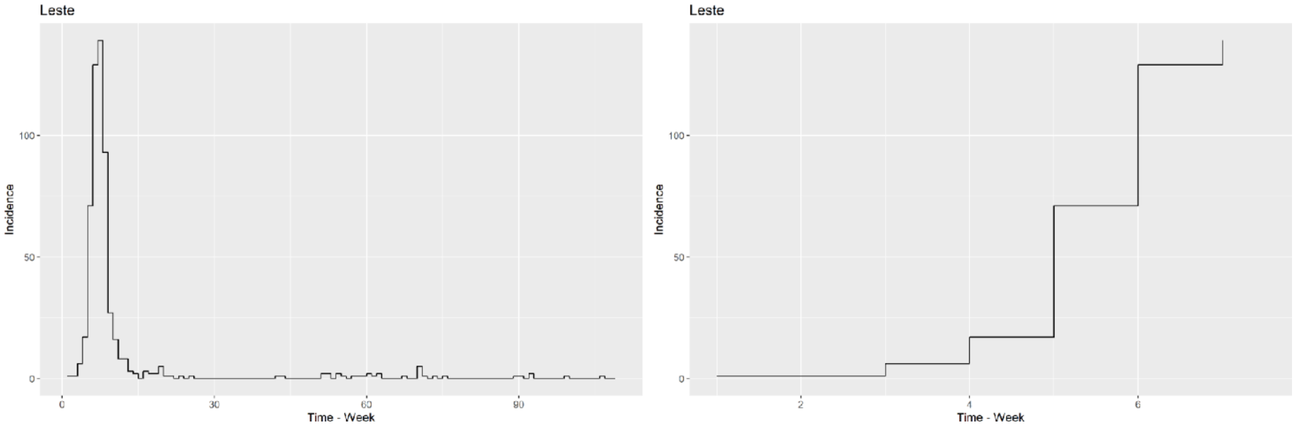

Figure D in S2 Text - Curve of reported cases for Macroregion Leste and the highlight for the exponential growth period

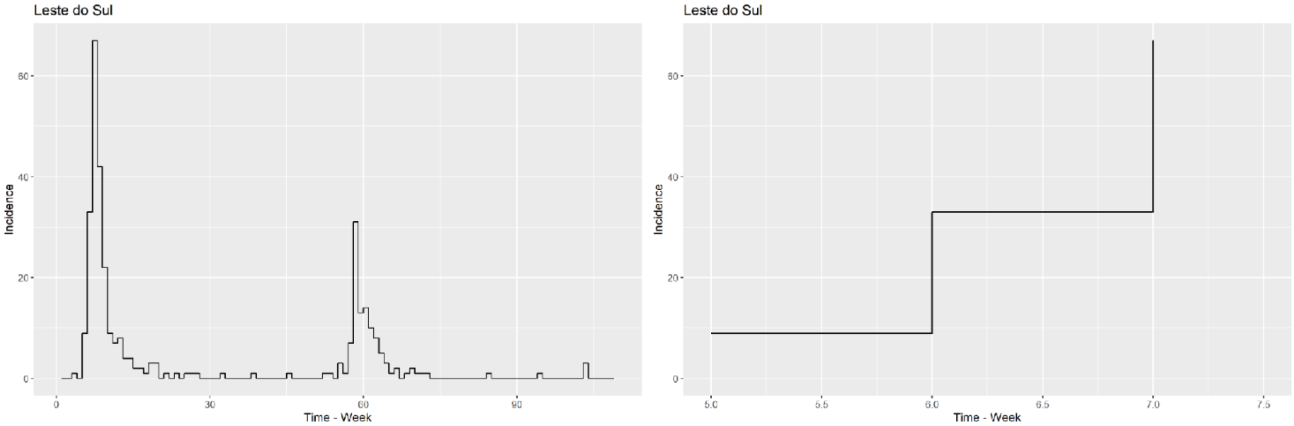

Figure E in S2 Text - Curve of reported cases for Macroregion Leste do Sul and the highlight for the exponential growth period

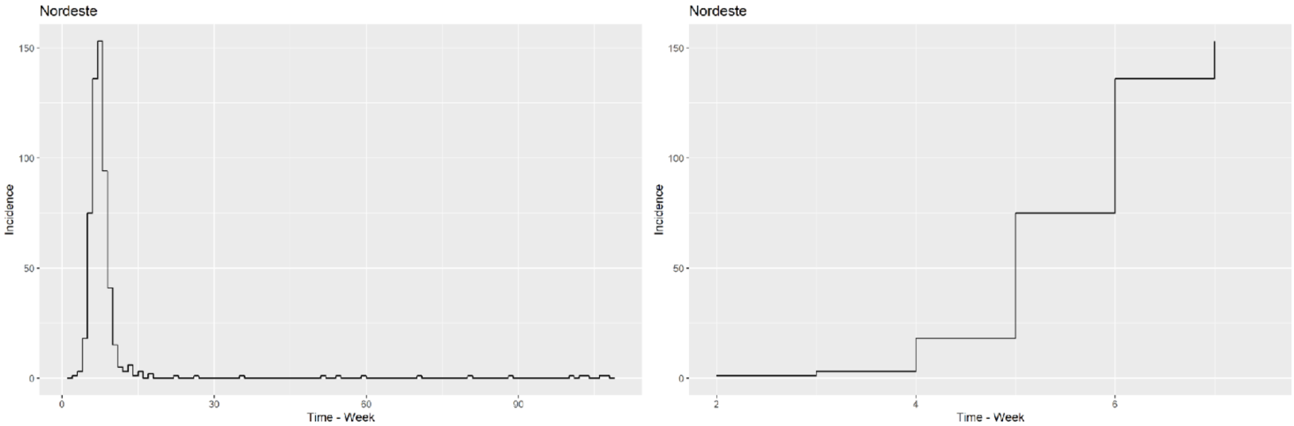

Figure F in S2 Text - Curve of reported cases for Macroregion Nordeste and the highlight for the exp onential growth period

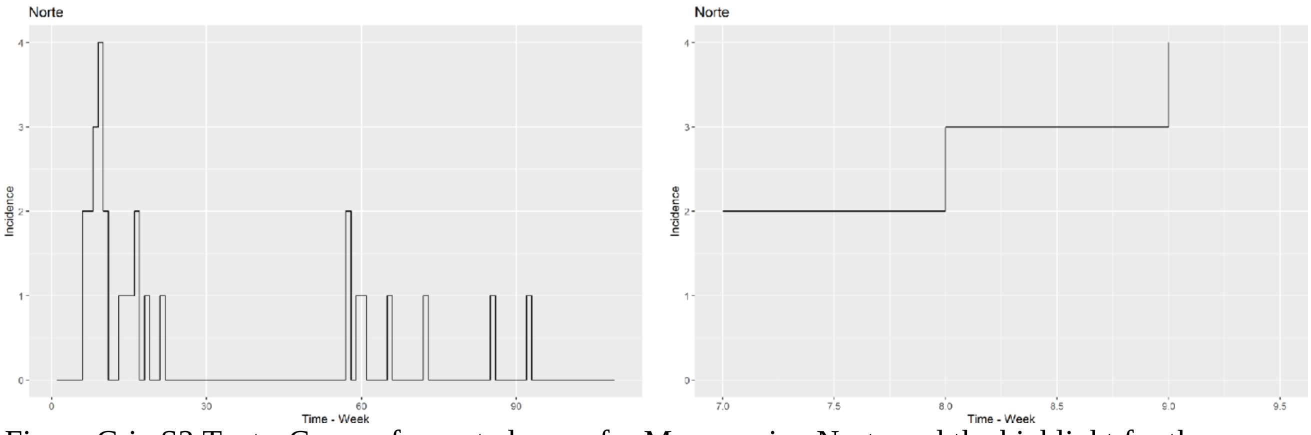

Figure G in S2 Text - Curve of reported cases for Macroregion Norte and the highlight for the expone ntial growth period

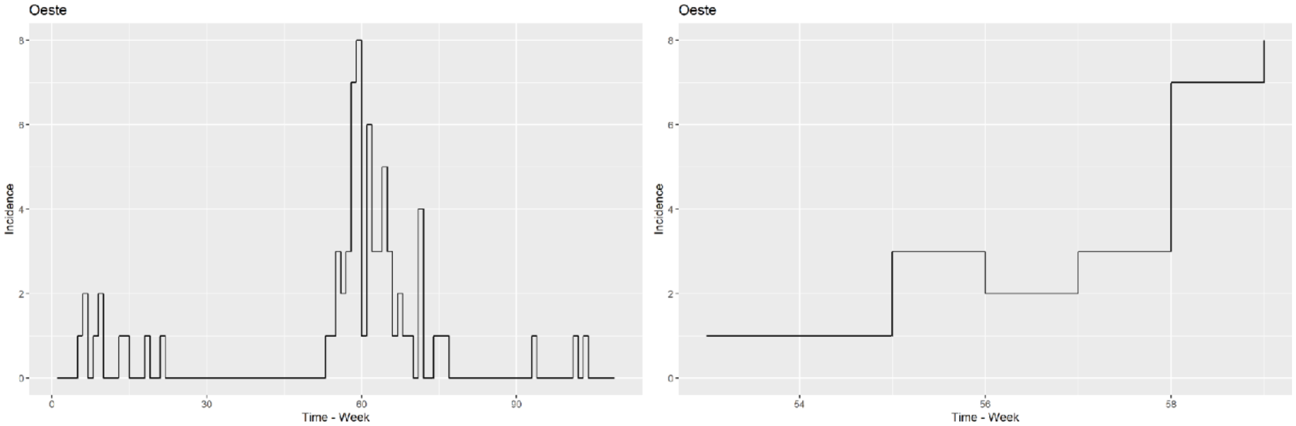

Figure H in S2 Text - Curve of reported cases for Macroregion Oeste and the highlight for the exponential growth period

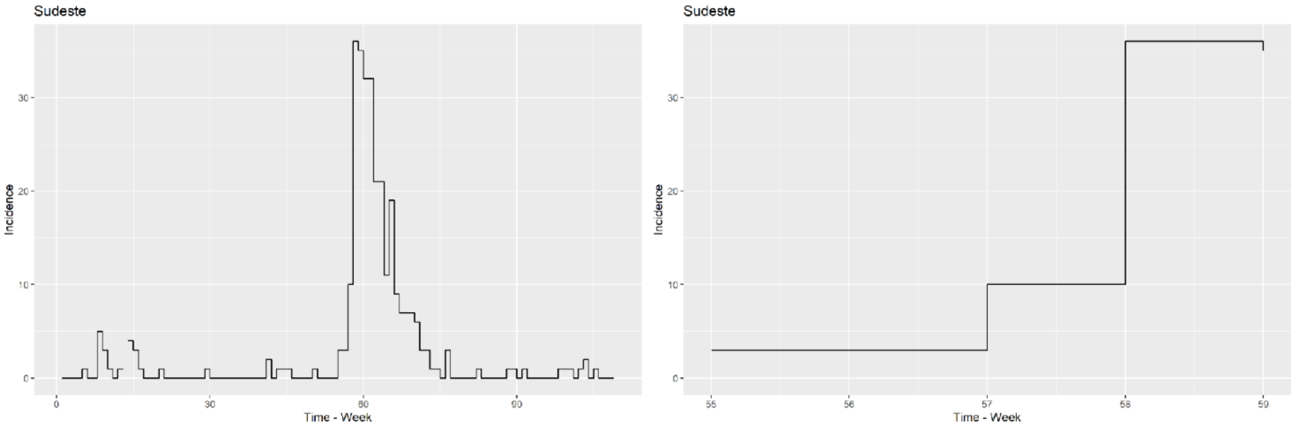

Figure I in S2 Text - Curve of reported cases for Macroregion Sudeste and the highlight for the exponential growth period

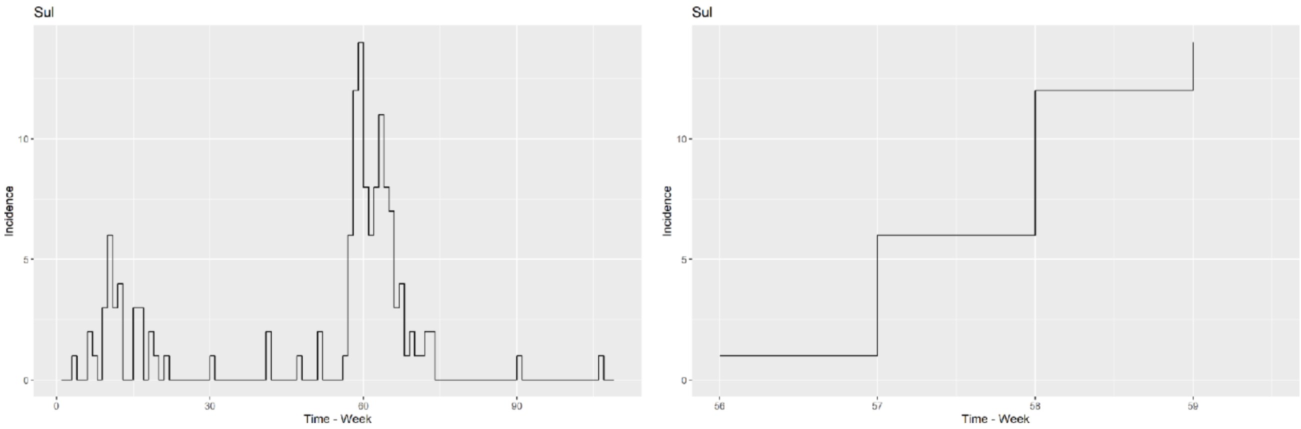

Figure J in S2 Text - Curve of reported cases for Macroregion Sul and the highlight for the exponential growth period
