# Supplementary material for: Occurrence of yellow fever outbreaks in a partially vaccinated population: An analysis of the effective reproduction number
Source: PLoS Negl Trop Dis. 2022 Sep 15;16(9):e0010741. doi: 10.1371/journal.pntd.0010741 (PMC9514630; doi:10.1371/journal.pntd.0010741)
Supplement: S3 Text — Fig A - 1st Wave of reported cases of yellow fever in the state of Minas Gerais and period selected to estimate reproductive number by the Methods EG and ML. Fig B—2nd Wave of reported cases of yellow fever in the state of Minas Gerais and period selected to estimate reproductive number by the Methods EG and ML. Fig C—Reported cases of yellow fever in the Macrorregion Centro state of Minas Gerais and period selected to estimate reproductive number by the methods EG and ML. Fig D—Reported cases of yellow fever in the Macrorregion Centro Sul state of Minas Gerais and period selected to estimate reproductive number by the methods EG and ML. Fig E—Reported cases of yellow fever in the Macrorregion Leste state of Minas Gerais and period selected to estimate reproductive number by the methods EG and ML. Fig F—Reported cases of yellow fever in the Macrorregion Leste do Sul state of Minas Gerais and period selected to estimate reproductive number by the methods EG and ML. Fig G—Reported cases of yellow fever in the Macrorregion Nordeste state of Minas Gerais and period selected to estimate reproductive number by the methods EG and ML. Fig H—Reported cases of yellow fever in the Macrorregion Norte state of Minas Gerais and period selected to estimate reproductive number by the methods EG and ML. Fig I—Reported cases of yellow fever in the Macrorregion Oeste state of Minas Gerais and period selected to estimate reproductive number by the methods EG and ML. Fig J—Reported cases of yellow fever in the Macrorregion Sudeste state of Minas Gerais and period selected to estimate reproductive number by the methods EG and ML. Fig L—Reported cases of yellow fever in the Macrorregion Sul state of Minas Gerais and period selected to estimate reproductive number by the methods EG and ML. Table A. Effective number of yellow fever reproduction according to estimation method (Exponential Growth e Maximum Likelihood), Minas Gerais, Brazil, 2016 to 2018. (PDF) [file pntd.0010741.s003.pdf]

Supplementary Material S3:

Occurrence of yellow fever outbreaks in a partly vaccinated population: an analysis of the effective reproduction number

Authors and affiliations
Fernanda Cristina da Silva Lopes Ferreira[1], Luiz Antônio Bastos Camacho[1], Daniel Antunes Maciel Villela[2*]

[1] National School of Public Health (ENSP), FIOCRUZ, Rio de Janeiro, Brazil
[2] Program of Scientific Computing (PROCC), FIOCRUZ, Rio de Janeiro, Brazil

Corresponding author. Email: daniel.villela@fiocruz.br

S3 Different methods of estimating the reproductive number

Figures A to L in S3 Text show the result of the analysis by the Methods EG [1] and ML [2] impleme nted by the *R0 Package* [3]

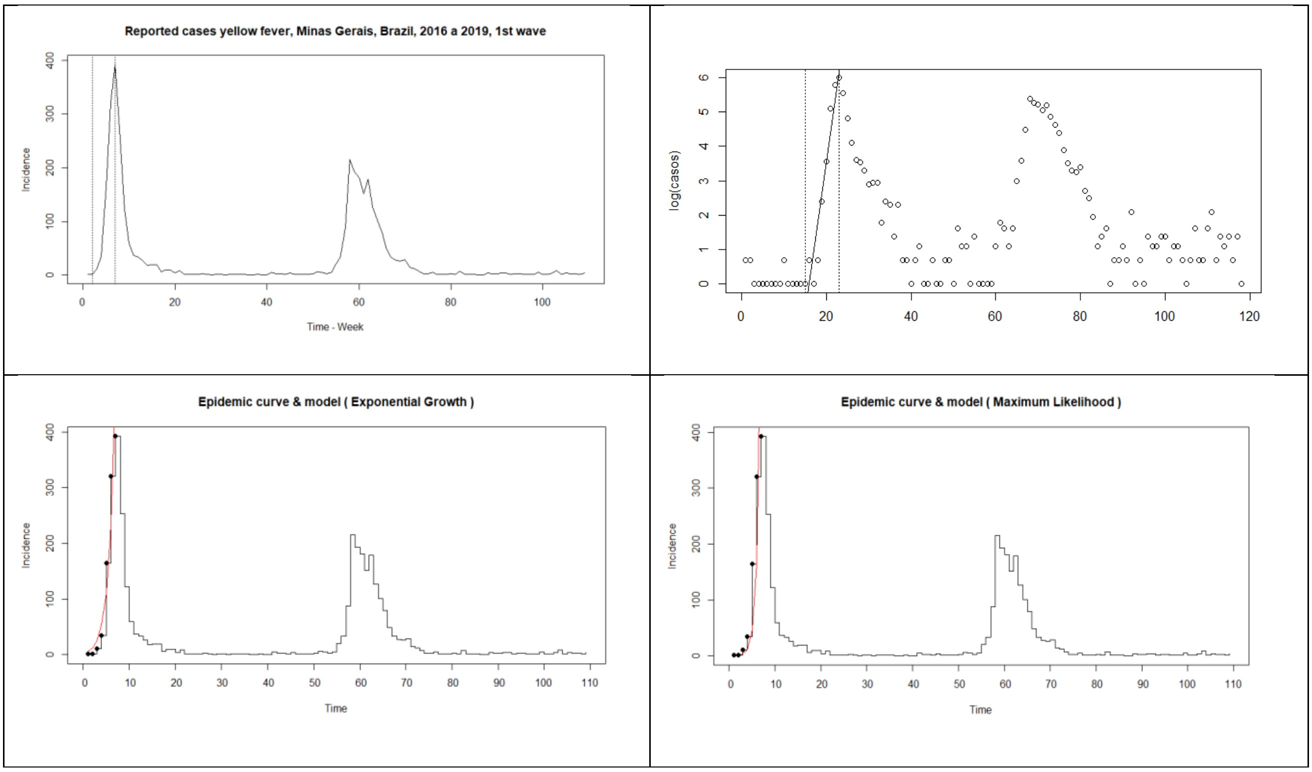

Figure A in S3 Text - 1st Wave of reported cases of yellow fever in the state of Minas Gerais and period selected to estimate reproductive number by the Methods EG and ML.

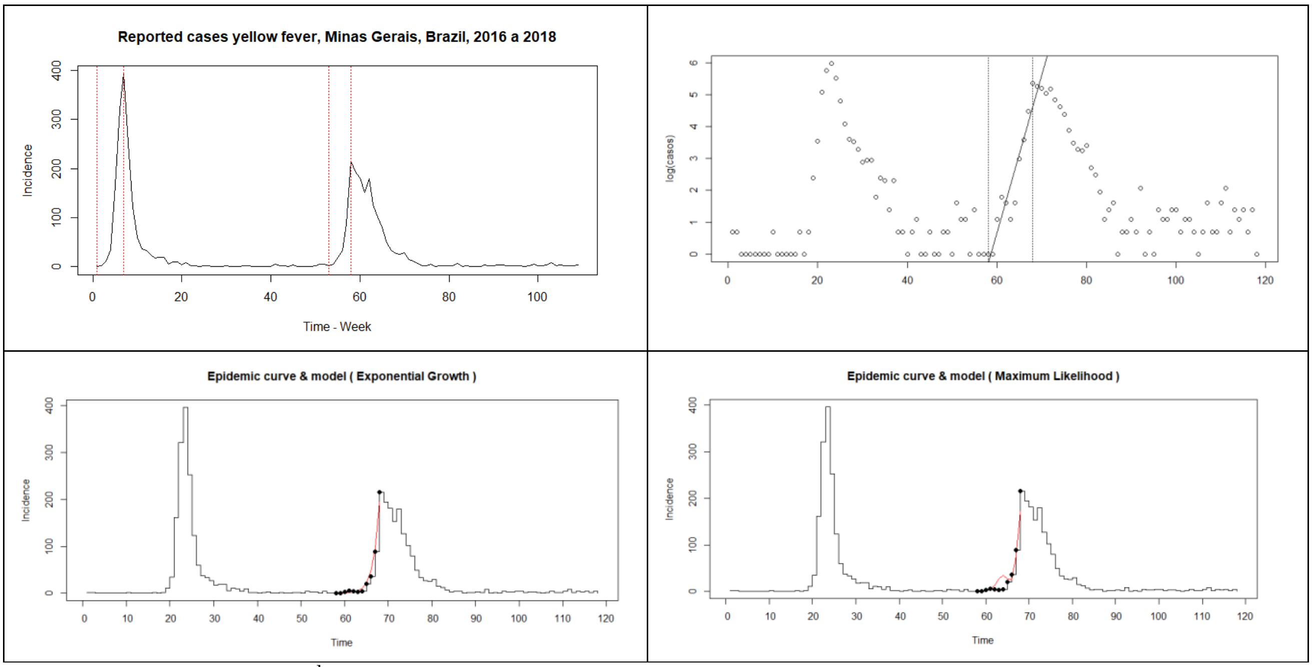

Figure B in S3 Text - 2nd Wave of reported cases of yellow fever in the state of Minas Gerais and period selected to estimate reproductive number by the Methods EG and ML.

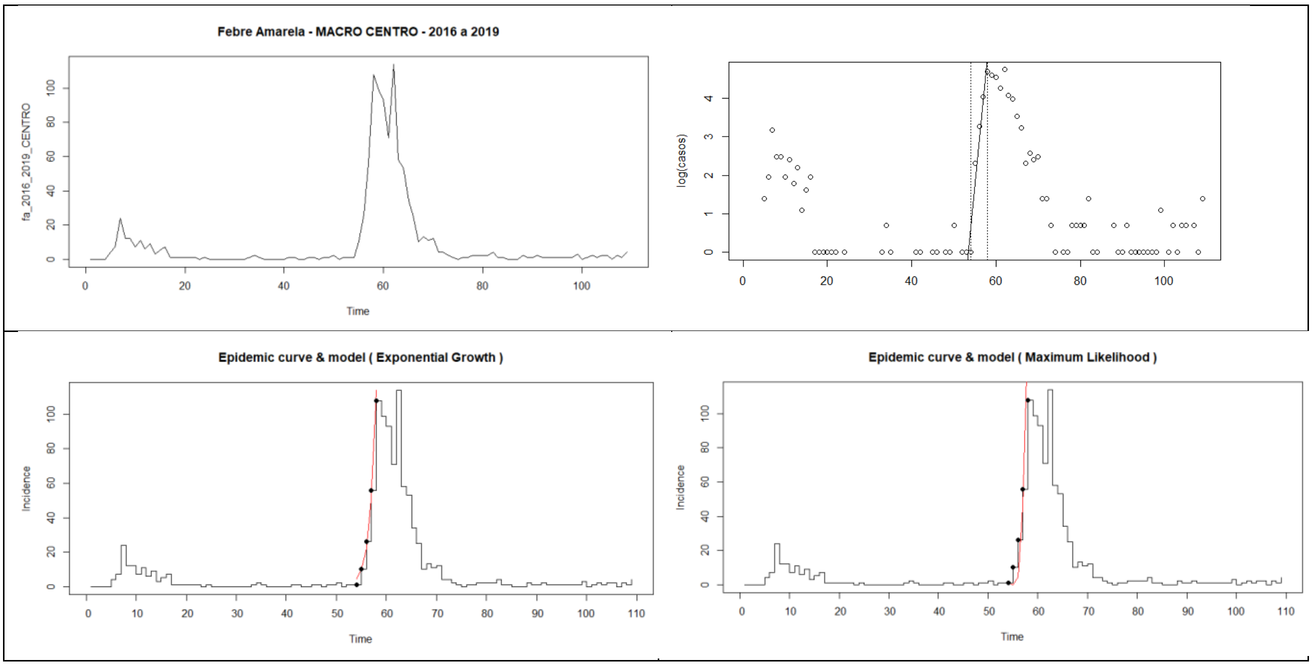

Figure C in S3 Text - Reported cases of yellow fever in the Macrorregion Centro state of Minas Gerais and period sel ected to estimate reproductive number by the methods EG and ML.

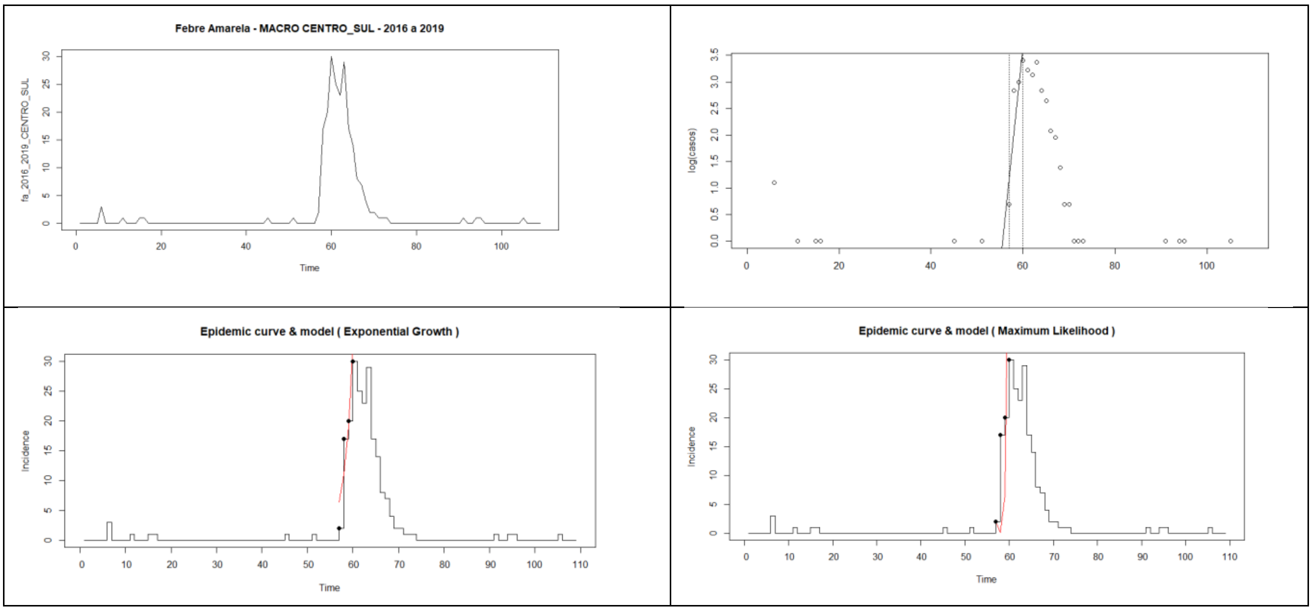

Figure D in S3 Text - Reported cases of yellow fever in the Macrorregion Centro Sul state of Minas Gerais and perio d selected to estimate reproductive number by the methods EG and ML.

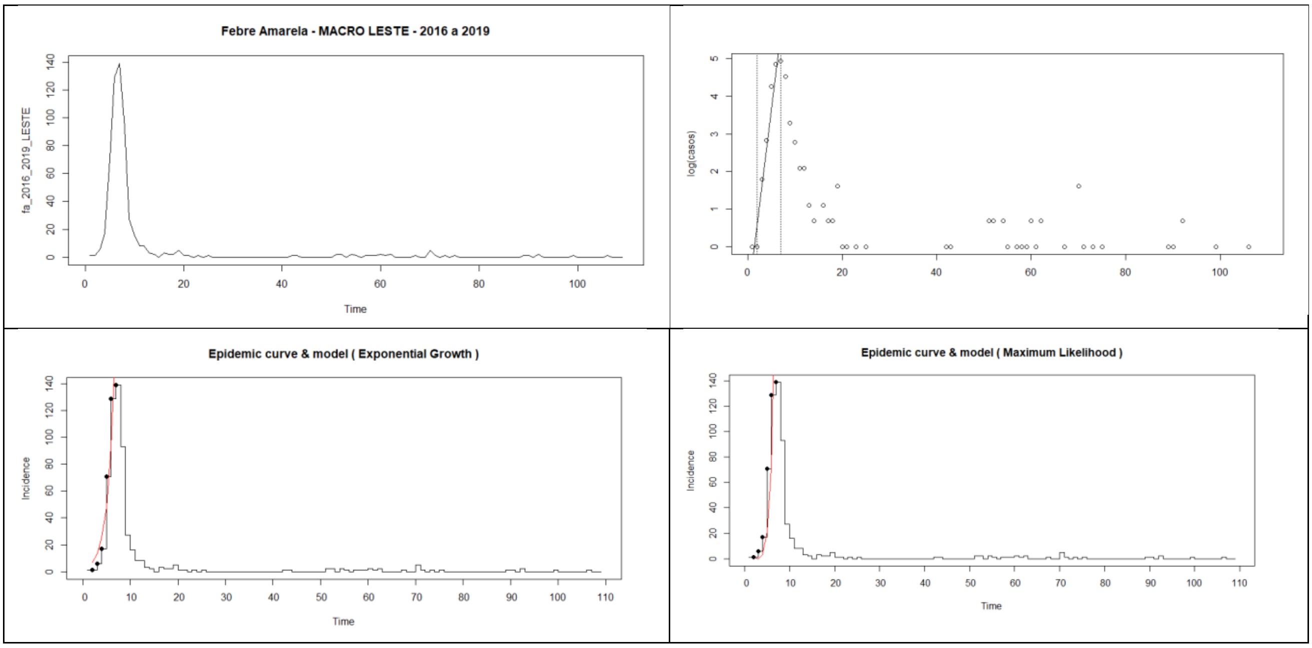

Figure E in S3 Text - Reported cases of yellow fever in the Macrorregion Leste state of Minas Gerais and period sele cted to estimate reproductive number by the methods EG and ML.

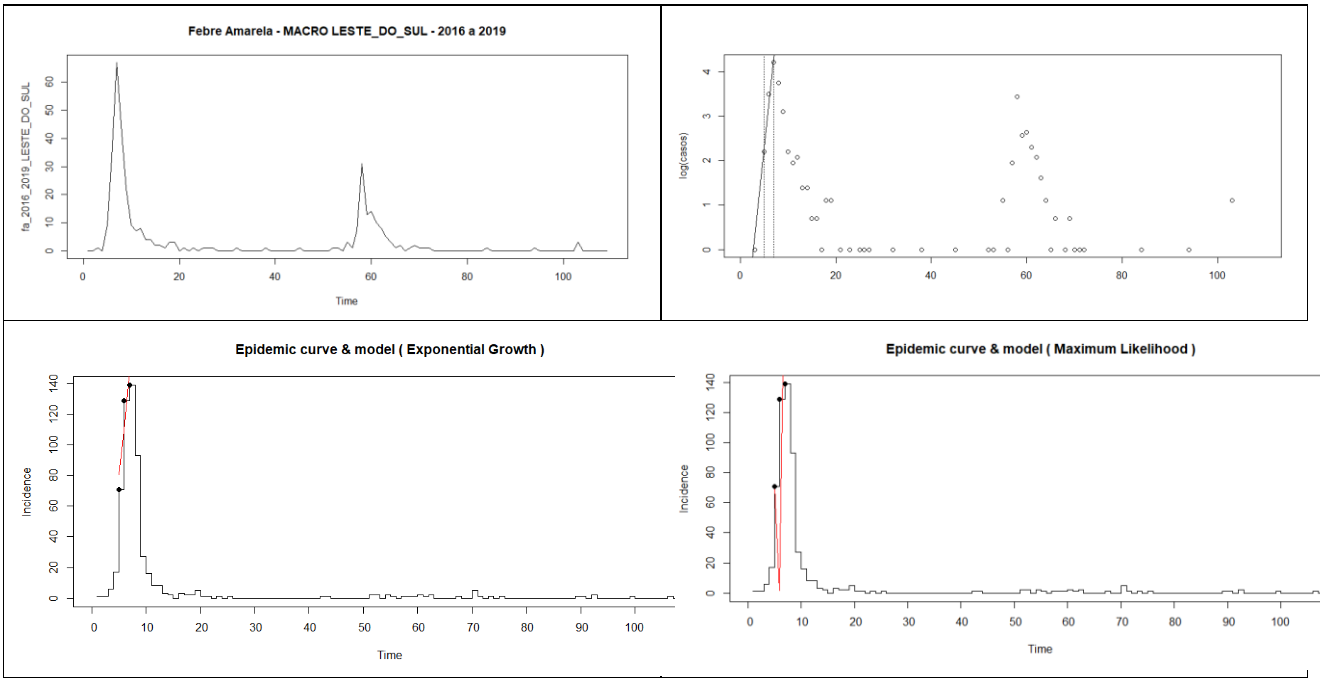

Figure F in S3 Text - Reported cases of yellow fever in the Macrorregion Leste do Sul state of Minas Gerais and peri od selected to estimate reproductive number by the methods EG and ML.

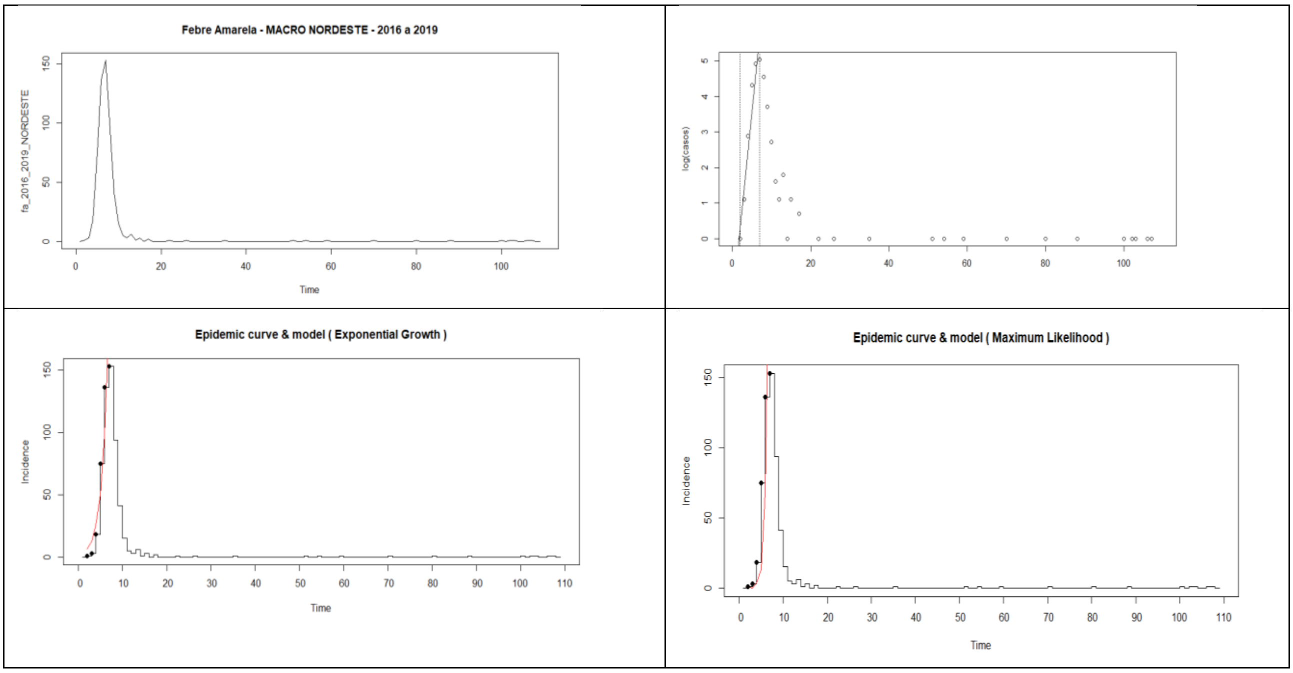

Figure G in S3 Text - Reported cases of yellow fever in the Macrorregion Nordeste state of Minas Gerais and period selected to estimate reproductive number by the methods EG and ML.

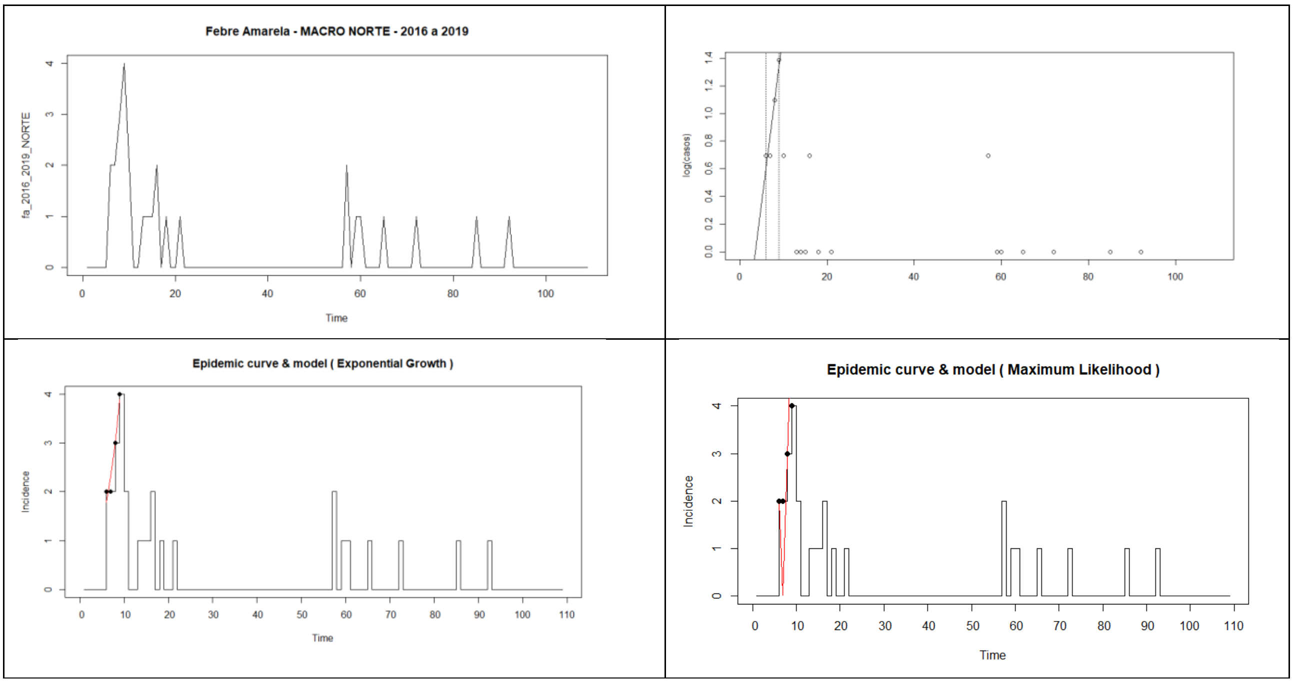

Figure H in S3 Text - Reported cases of yellow fever in the Macrorregion Norte state of Minas Gerais and period sel ected to estimate reproductive number by the methods EG and ML.

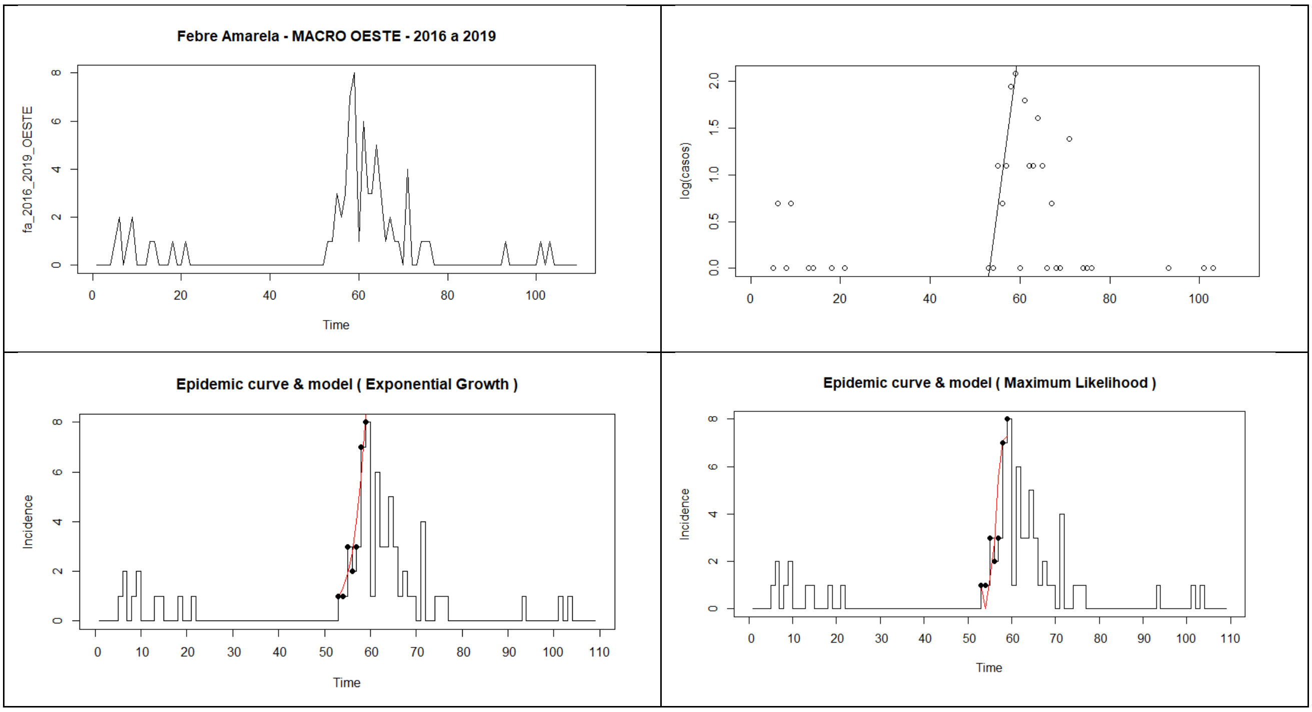

Figure I in S3 Text - Reported cases of yellow fever in the Macrorregion Oeste state of Minas Gerais and period sele cted to estimate reproductive number by the methods EG and ML.

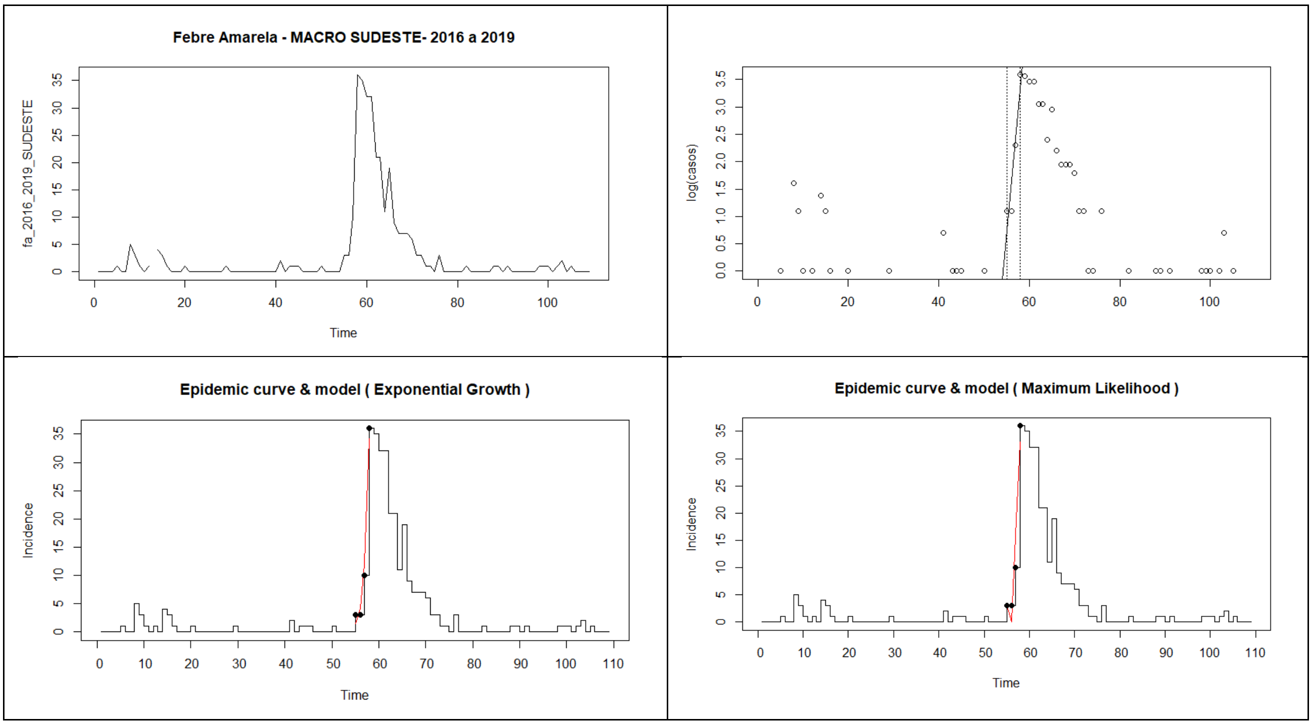

Figure J in S3 Text - Reported cases of yellow fever in the Macrorregion Sudeste state of Minas Gerais and period se lected to estimate reproductive number by the methods EG and ML.

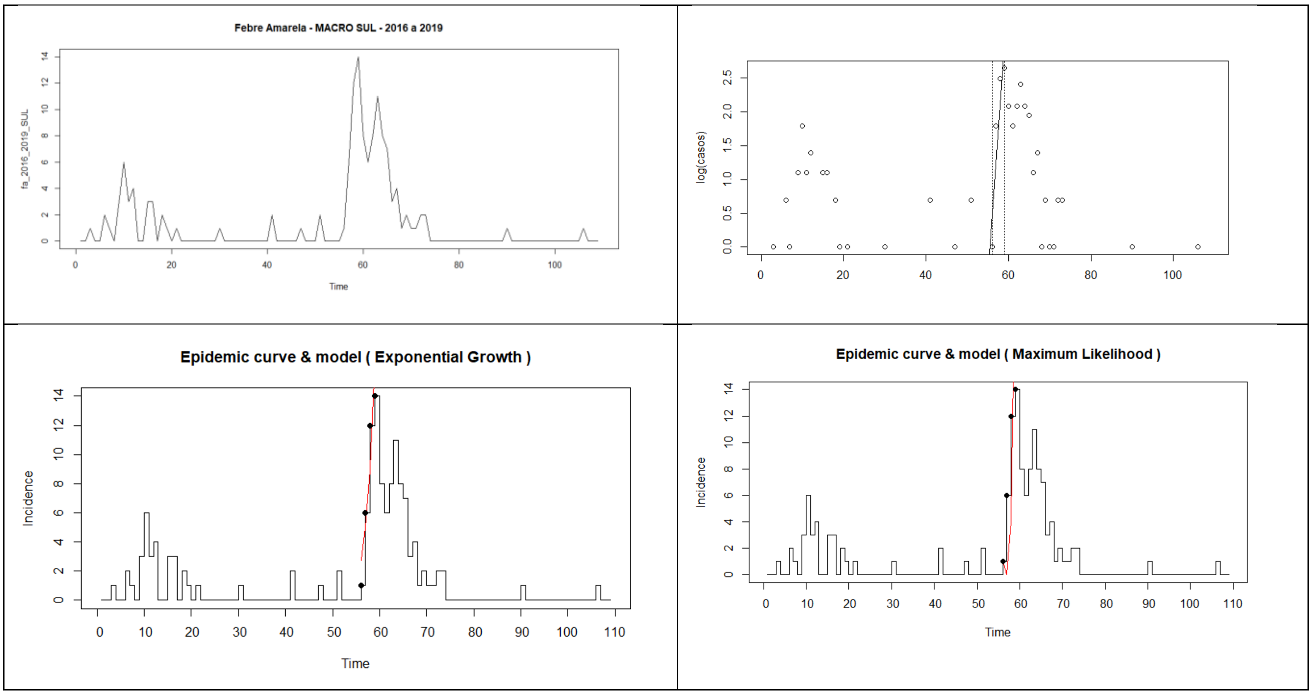

Figure L in S3 Text - Reported cases of yellow fever in the Macrorregion Sul state of Minas Gerais and period select ed to estimate reproductive number by the methods EG and ML.

Table A in S3 Text. Effective number of yellow fever reproduction according to estimation meth od
(Exponential Growth e Maximum Likelihood), Minas Gerais, Brazil, 2016 to 2018.
Table S3 shows the estimated values for the playback number according to the EG or ML method i mplemented by *Package R0*. Highlight values refer to statistically significant estimated values accor ding to the regression applied to the case aggregation window that determined the exponential growt h period.

Local	Estimation Methods	
	EG*	ML**
MG 1st wave	**8.74 [7.55, 10.14]**	**7.68 [6.10,8.40]**
2nd wave	**8.05 [6.60, 9.95]**	**6.42 [5.55,7.37]**
**Centro**	**10.89 [7.47, 16.18]**	**8.88 [7.25, 10.74]**
Centro Sul	5.08 [2.59, 10.18]	6.90 [4.82, 9.51]
**Leste**	**6.85 [5.43, 8.71]**	**6.48 [5.58, 7.47]**
Leste do Sul	13.80 [6.53, 29.97]	23.86 [17.84, 31.10]
**Nordeste**	**7.42 [5.90, 9.41]**	**6.91 [5.98, 7.94]**
Norte	2.20 [0.42, 11.49]	3.19 [1.07, 7.10]
**Oeste**	**3.01 [1.57, 6.1]**	**2.90 [1.55, 4.86]**
**Sudeste**	**20.29 [8.13, 55.72]**	**11.54 [7.55, 16.73]**
Sul	5.91 [2.22, 16.69]	8.60 [5.05, 13.54]

*Exponential Growth; **Maximum Likelihood

References

1. Wallinga J, Lipsitch M. How generation intervals shape the relationship between growth rates and reproductive numbers. Proc R Soc B Biol Sci. 2007;274: 599–604. doi:10.1098/rspb.2006.3754

2. White LF, Wallinga J, Finelli L, Reed C, Riley S, Lipsitch M, et al. Estimation of the reproductive number and the serial interval in early phase of the 2009 influenza A/H1N1 pandemic in the USA. Influenza Other Respir Viruses. 2009;3: 267–276. doi:10.1111/j.1750-2659.2009.00106.x

3. Obadia T, Haneef R, Boëlle P-Y. The R0 package: a toolbox to estimate reproduction numbers for epidemic outbreaks. BMC Med Inform Decis Mak. 2012;12: 147. doi:10.1186/1472-6947-12-147
